# Ambiphilic Al−Cu Bonding

**DOI:** 10.1002/anie.202104658

**Published:** 2021-05-20

**Authors:** Han‐Ying Liu, Ryan J. Schwamm, Michael S. Hill, Mary F. Mahon, Claire L. McMullin, Nasir A. Rajabi

**Affiliations:** ^1^ Department of Chemistry University of Bath Bath BA2 7AY UK

**Keywords:** aluminum, coordination modes, copper, ligand effects, metal–metal interactions

## Abstract

Copper‐alumanyl complexes, [LCu‐Al(SiN^Dipp^)], where L=carbene=NHC^iPr^ (N,N′‐diisopropyl‐4,5‐dimethyl‐2‐ylidene) and ^Me2^CAAC (1‐(2,6‐diisopropylphenyl)‐3,3,5,5‐tetramethyl‐pyrrolidin‐2‐ylidene) and featuring unsupported Al−Cu bonds, have been prepared. Divergent reactivity observed with carbodiimides and CO_2_ implies an ambiphilicity in the Cu–Al interaction that is dependent on the identity of the carbene co‐ligand.

## Graphical abstract

Cu(ts) both ways: Carbene‐supported copper‐alumanyl complexes, [LCu‐Al(SiN^Dipp^)], display divergent reactivity with carbodiimides and CO_2_, implying an ambiphilicity in the Cu–Al interaction that is dependent on the identity of the carbene co‐ligand.

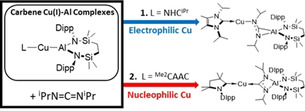

The pursuit of unsupported metal‐metal bonds has long provoked theoretical curiosity and continues to motivate some of the most striking advances in synthetic chemistry.[^1^, ^2^] Aldridge and Goicoechea's landmark report of the potassium alumanyl, [K{Al(NON)}]_2_ (**I**, where NON is the chelating tridentate ligand 4,5‐bis(2,6‐diisopropylanilido)‐2,7‐di‐*tert*‐butyl‐9,9‐dimethylxanthene) has spawned a variety of related species,[^3^, ^4^, ^5^, ^6^, ^7^, ^8^, ^9^] which have demonstrated their value as potent sources of nucleophilic aluminium and have been used to access several unprecedented Al−M bonded molecules.[^10^, ^11^, ^12^] For example, the reaction of **I** with ^*t*^Bu_3_PAuI gave rise to the two‐coordinate gold complex, [(NON)AlAuP^*t*^Bu_3_] (**II**).^[12]^ Consistent with the expectation presented by the relative Pauling electronegativities of the constituent metals (Au: 2.54; Al: 1.61), theoretical assessment indicated the Al‐Au interaction in **II** is significantly polarized, that is, Au^δ−^‐Al^δ+^. Furthermore, the implication that compound **II** could act as a nucleophilic source of gold was validated by its reaction with *N*,*N′*‐diisopropylcarbodiimide and CO_2_ to provide the respective Au−C bonded insertion products, [(NON)Al(X_2_C)AuP^*t*^Bu_3_] (X=N^*i*^Pr, **III**; X=O, **IV**, Scheme 1). The related boryl gold complex, [(IPr)Au‐B(*o*‐tol)_2_] (**V,** IPr=*N*,*N*′‐bis(2,6‐diiso‐propylphenyl)imidazol‐2‐ylidene), in which interaction with a diarylboryl substituent induces similar Au^δ−^‐B^δ+^ polarization, was subsequently reported by Yamashita and co‐workers (Scheme 1).^[13]^ Relativistic contraction of the 6s orbital results in the highest electron affinity of any transition metal (2.30 eV), while the quasi‐closed shell 5*d* ^10^6*s* ^2^ configuration resulting from its reduction dictates that gold is the sole transition metal to give rise to a stable “naked” (auride, Au^−^) monoanion in the condensed phase.^[14]^ These attributes do not extend to gold's lighter Group 11 congeners, such that the induction of analogous nucleophilic character at either silver (1.30 eV) or copper (1.23 eV) would appear to be even more challenging.^[15]^

**Scheme 1:**
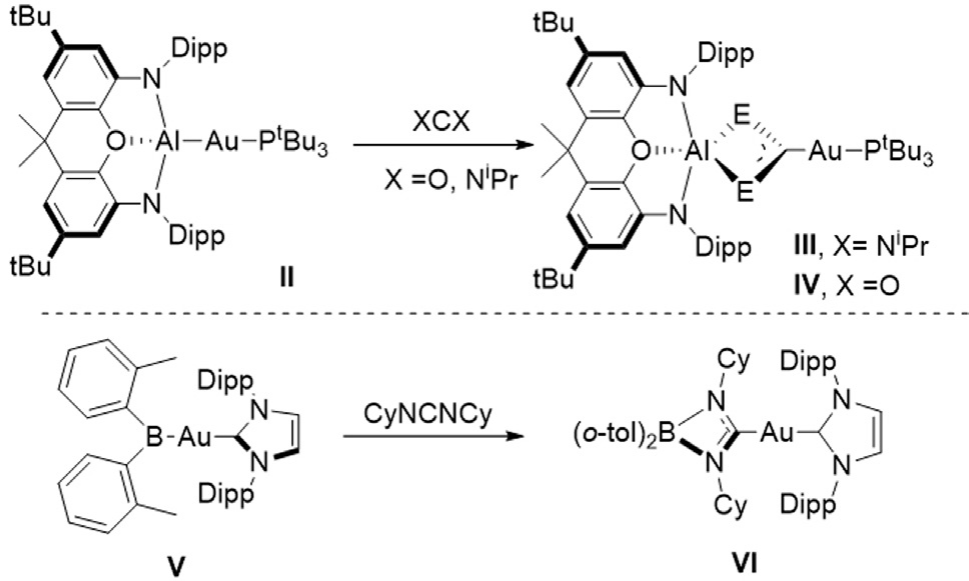
Examples of nucleophilic gold molecules.

A variety of terminal copper boryl species have been described since Sadighi's initial demonstration that the two‐coordinate NHC derivative, [(IPr)CuBpin] (**VII**, pin=pinacol), can perform the stoichiometric and, in the presence of B_2_pin_2_, catalytic reduction of CO_2_ to CO (Scheme 2).[^16^, ^17^, ^18^, ^19^, ^20^, ^21^, ^22^, ^23^, ^24^] None of these species, however, has been identified as a source of nucleophilic copper. Indeed, this prospect has been explicitly excluded by DFT analysis of both the deoxygenative reactivity shown in Scheme 2 and related Cu/B addition to carbonyl‐ and imine‐containing small molecules.[^25^, ^26^, ^27^, ^28^]

**Scheme 2:**
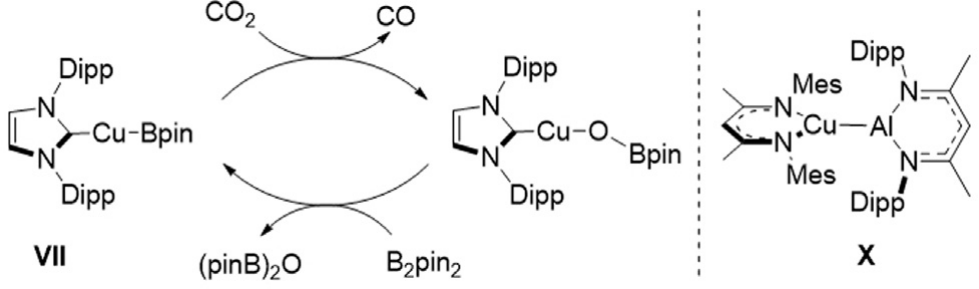
Catalytic reduction of CO_2_ by [(IPr)CuBpin] (**VII**) and the structure of **X**.

Heavier group 13 species featuring unsupported Cu−M bonding are limited to three compounds containing direct copper‐aluminium interactions and several reports of copper gallyl complexes.[^29^, ^30^, ^31^, ^32^, ^33^] Early work by Fischer and co‐workers’ utilised [Cp*Al]_4_ for the synthesis of the cluster derivatives, [(Cp*AlCu)_6_H_4_] (**VIII**) and [Cu_43_Al_12_(Cp*)_12_] (**IX**).[^31^, ^32^] More recently, Power and co‐workers have described [{(^Mes^BDI)Cu‐Al(^Dipp^BDI)}] (**X**, Scheme 2, ^Mes^BDI=*N*,*N*′‐bis(2,4,6‐trimethylphenyl)pentane‐2,4‐diiminate; ^Dipp^BDI=*N*,*N*′‐bis(2,6‐diisopropylphenyl)pentane‐2,4‐diiminate), which features a terminal Cu−Al bond.^[33]^ DFT analysis of **X** indicated that approximately 50 % of the calculated association enthalpies could be attributed to London dispersion forces between the *N*‐aryl substituents, while the calculated orbital component consisted primarily of σ‐type donation from Al to Cu. Although further reactivity is yet to be described, these observations imply that the Al−Cu bond in **X** is best considered as an Al:→Cu dative interaction. In this contribution we report the first examples of X‐type alumanyl copper complexes.

Inspired by the synthesis of compound **I**, we have recently described the seven‐membered heterocyclic potassium diamidoalumanyl species, [K{Al(SiN^Dipp^)}]_2_ (**XI**, SiN^Dipp^={CH_2_SiMe_2_NDipp}_2_ Scheme 3).^[8]^ With the above observations in mind, **XI** was reacted with the Cu^I^ chloride carbene adducts, [(NHC^*i*Pr^)CuCl] and [(^Me2^CAAC)CuCl] (NHC^*i*Pr^=*N*,*N*′‐diisopropyl‐4,5‐dimethyl‐2‐ylidene; ^Me2^CAAC=1‐(2,6‐diisopropylphenyl)‐3,3,5,5‐tetramethylpyrrolidin‐2‐ylidene), yielding the Cu−Al bonded complexes [LCu‐Al(SiN^Dipp^)] (L=NHC^*i*Pr^ (**1**); ^Me2^CAAC (**2**)) in good yields after work‐up (Scheme 3).

**Scheme 3:**
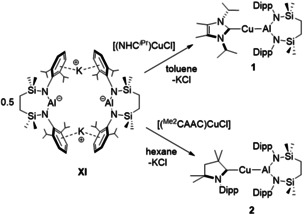
Syntheses of carbene‐stabilized copper‐alumanyl complexes **1** and **2**.

The ^1^H NMR spectra of **1** and **2** are consistent with a 1:1 ratio of the carbene to SiN^Dipp^ ligands, while the ^13^C NMR spectra display resonances at *δ*=175.9 and 254.2 ppm attributed to the respective carbene Cu‐*C* environments. The solid‐state structures of **1** and **2** were determined through single crystal X‐ray diffraction analysis, confirming the formation of copper alumanyl complexes (Figure 1). Both compounds comprise a two‐coordinate copper atom with C‐Cu‐Al angles subtended by the carbene and Al(SiN^Dipp^) ligands that approach linearity [C‐Cu‐Al: 178.85(4) (**1**); 173.42(6)° (**2**)]. Both the Cu1−C31 [1.9529(12) Å (**1**); 1.964(2) Å (**2**)] and Cu1−Al1 [2.3449(4) Å (**1**); 2.4028(7) Å (**2**)] distances in compound **2** are longer than those of **1**, most likely a consequence of increased steric pressure in **2**. In both cases, the Cu−Al bonds are comparable to the shortest Cu‐Al interaction observed in the cluster species **VIII** and **IX** [range: 2.4027(14) to 2.7189(14) Å], but are notably longer than the terminal Al−Cu bond of compound **X** [2.3010(6) Å].^[31]^ This latter feature is attributed to the transoid disposition of the strongly binding carbene ligands.

**Figure 1:**
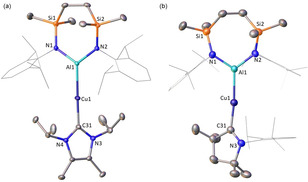
Displacement ellipsoid plot of a) compound **1** and b) compound **2** (30 % probability ellipsoids). Dipp substituents are shown as wireframe and hydrogen atoms are omitted for clarity. Selected bond lengths [Å] and angles [°]; **1**: Cu1‐Al1 2.3449(4), Cu1‐C31 1.9529(12), Al1‐N1 1.8455(10), Al1‐N2 1.8473(10), C31‐Cu1‐Al1 178.85(4), N1‐Al1‐N2 112.05(5), N1‐Al1‐Cu1 123.41(4), N2‐Al1‐Cu1 124.54(3); **2**: Cu1‐Al1 2.4028(7), Cu1‐C31 1.964(2), Al1‐N1 1.8668(18), Al1‐N2 1.8546(18), C31‐Cu1‐Al1 173.42(6), N1‐Al1‐N2 110.96(8).

To provide experimental insight into the nature of the Cu−Al bonds, the copper alumanyl derivatives were reacted with heteroallenes. Reactions of **1** and **2** with *N*,*N′*‐diisopropylcarbodiimide each resulted in the gradual consumption of the starting materials and formation of single new species, **3** and **4**, respectively, which were isolated in good yields (≥70 %) after work‐up (Scheme 4). The ^1^H NMR spectra of **3** and **4** both show broadened resonances corresponding to the SiN^Dipp^ ligand, consistent with restrictions in conformation. The ^*i*^Pr methine resonances of the former carbodiimide fragment in **3** are separated into two distinct signals at *δ*=3.32 and 4.38 ppm. In contrast, the analogous protons in **4** appear as a single sharp resonance at *δ*=3.37 ppm. Although the NHC donor carbon of **3** could not be observed, the ^13^C NMR spectrum of **4** was characterized by the appearance of a low field resonance at *δ*=220.9 ppm arising from the copper‐coordinated carbon center.

**Scheme 4:**
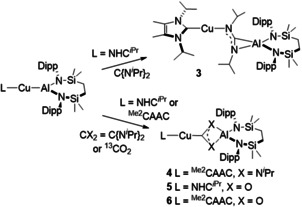
Reaction of copper‐alumanyl complexes **1** and **2** with *N*,*N′*‐diisopropylcarbodiimide and ^13^CO_2_.

The reaction of **1** and **2** with ^13^CO_2_ resulted in the rapid formation of the copper metallacarboxylate species, **5** and **6**, respectively. The ^1^H NMR spectra of **5** and **6** show a single set of resonances for each of the carbene and SiN^Dipp^ ligands. The ^13^C NMR spectra show isotopically‐enriched resonances at *δ*=236.2 (**5**) and 234.9 (**6)** ppm, suggesting closely related structures. These low field signals are characteristic of Cu−*C*O_2_ bonding and are comparable to diagnostic resonances observed in the related gold(I) metallacarboxylate (**IV**, *δ*=242.3 ppm).^[11]^

Compounds **3**–**6** displayed excellent thermal stability, with no evidence of degradation or isomerization when heated to 60 °C for 3 days. In addition, the CO_2_ fragment is retained in **5** and **6**, contrasting the copper boryl‐mediated decarbonylation of CO_2_ summarised in Scheme 2. While attempts to obtain suitable crystals of **5** were unsuccessful, single crystal X‐ray diffraction analysis of **3**, **4** and **6** confirmed insertion of the heteroallene into the Cu−Al bonds (Figures 2 and 3). The arrangement of the central *μ*‐CN_2_ fragment in **3** and **4**, however, differs between the two species. The solid‐state structure of **3** features a two‐coordinate copper center, ligated by NHC^*i*Pr^ and a single nitrogen atom of the CN_2_ fragment [Cu−C31 1.8959(18) Å; Cu−N6 1.8846(15) Å]. The coordination sphere of the aluminium is satisfied by a side‐on *η* ^*2*^‐interaction with the C42−N5 bond of the {CN_2_} unit, resulting in the formation of a constrained three‐membered AlCN metallacycle with Al−C, Al−N and C−N distances of 1.9554(17), 1.8693(14) and 1.358(2) Å, respectively. In contrast, compound **4** crystallizes as the cupra‐amidinate, with the ^Me2^CAAC‐ligated copper center bound to the {CN_2_} fragment through the central carbon atom in an analogous manner to that observed in the gold derivative **III** (Scheme 1). The Cu1−C51 distance [1.960(3) Å] is longer than the carbenic Cu1−Cu31 interaction [1.919(3) Å]. The cupra‐amidinate coordinates the aluminium center in a *N*,*N′*‐bidentate fashion, with essentially identical Al−N distances [Al1−N4 1.908(2); Al1−N5 1.923(2) Å].

**Figure 2:**
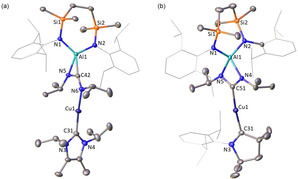
Displacement ellipsoid plot of a) compound **3** and b) compound **4** (30 % probability ellipsoids). Dipp substituents are shown as wireframe and hydrogen atoms are omitted for clarity. Selected bond lengths [Å] and angles [°]; **3**: Cu1‐N6 1.8846(15), Cu1‐C31 1.8959(18), Al1‐N1 1.8425(14), Al1−N2 1.8411(14), Al1‐N5 1.8693(14), Al1‐C42 1.9554(17), N6‐Cu1‐C31 174.70(7), N1‐Al1‐N5 115.08(6); **4**: Cu1‐C31 1.919(3), Cu1‐C51 1.960(3), Al1‐N1 1.860(2), Al1‐N2 1.862(2), Al1‐N4 1.908(2), Al1‐N5 1.923(2), C31‐Cu1‐C51 173.82(13).

**Figure 3:**
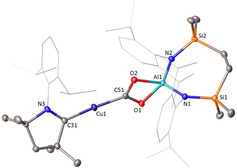
Displacement ellipsoid plot of compound **6** (30 % probability ellipsoids). Dipp substituents are shown as wireframe and hydrogen atoms are omitted for clarity. Selected bond lengths [Å] and angles [°]; Cu1‐C31 1.894(2), Cu1‐C51 1.902(2), Al1‐O1 1.8563(16), Al1‐O2 1.8405(17), Al1‐N1 1.8096(19), Al1‐N2 1.8125(18), C31‐Cu1‐C51 171.16(10), O2‐Al1‐O1 71.34(7).

The solid‐state structure of **6** confirms the formation of a cupra‐carboxylate species, with a closely related structure to **4**. The ^Me2^CAAC‐coordinated copper center bonds to the central *μ*‐CO_2_ unit through the central carbon atom, while the aluminium center is chelated by the two oxygen atoms. In a similar fashion to that of **4,** the Cu1−C51 distance [1.902(2) Å] is long and essentially identical to the carbenic Cu1−C31 bond [1.894(2) Å]. The Al−O [1.8563(16) and 1.8405(17) Å] and C−O distances [1.301(3), 1.307 (3) Å] are consistent with delocalisation of the π‐electron density over the entirety of the {CO_2_} fragment.

The contrast in reaction products obtained from insertion of *N*,*N′*‐diisopropylcarbodiimide into the Cu−Al bond of **1** and **2** suggests an adjustment in the apparent polarity of the bond upon changing the carbene donor. Further insight into the structures of **1** and **2** was provided by DFT calculations (See SI for details). Both optimized to geometries close to those observed in the solid state, albeit with slightly overestimated Cu−Al bond lengths [2.383 (**1**); 2.431 Å (**2**)]. Calculated Wiberg bond indices [0.868 (**1**); 0.806 (**2**)] were commensurate with a significant degree of covalency between the two atoms, a viewpoint reinforced by the relative contributions of both metals to the localised Al−Cu bonding orbitals [56.5 % on Al and 43.5 % on Cu (**1**); 60 % on Al and 40 % on Cu (**2**)]. Although variation of the carbene donors induced adjustments to the concomitant NBO charges, [*q*_Al_ +1.28, *q*_Cu_ −0.09 (**1**); *q*_Al_ +1.215, *q*_Cu_ +0.72 a.u. (**2**)], both sets of data invoke similar Al^δ+^‐Cu^δ−^ polarization across the metal‐metal bonds.

In conclusion, two‐coordinate copper alumanyl derivatives are readily accessible by salt elimination between a potassium alumanyl anion and carbene‐ligated copper(I) chloride. Initial studies of the reactivity of the Al−Cu bonds implicate the installation of nucleophilic character at the copper center. This behaviour, however, is apparently modulated, either by variation of the carbene co‐ligand basicity or adjustment of the electrophilic heteroallene reaction partner. We are continuing to study these effects on the reactivity of copper and its heavier Group 11 congeners.

Supporting information for this article is given via a link at the end of the document and in CCDC 2073091, 2073092, 2073093, 2073094 and 2073095.

## Conflict of interest

The authors declare no conflict of interest.

## Supporting information

As a service to our authors and readers, this journal provides supporting information supplied by the authors. Such materials are peer reviewed and may be re‐organized for online delivery, but are not copy‐edited or typeset. Technical support issues arising from supporting information (other than missing files) should be addressed to the authors.

Supplementary

## References

[1] M. J. Taylor , Metal-to-Metal Bonded States of the Main Group Elements, Academic Press, London, 1975.

[2] J. F. Berry , C. C. Lu , Inorg. Chem. 2017, 56, 7577–7581. 28715854 10.1021/acs.inorgchem.7b01330

[3] J. Hicks , P. Vasko , J. M. Goicoechea , S. Aldridge , Nature 2018, 557, 92–95. 29662211 10.1038/s41586-018-0037-y

[4] J. Hicks , P. Vasko , J. M. Goicoechea , S. Aldridge , Angew. Chem. Int. Ed. 2021, 60, 1702–1713; 10.1002/anie.202007530 32567755

[5] R. J. Schwamm , M. D. Anker , M. Lein , M. P. Coles , Angew. Chem. Int. Ed. 2019, 58, 1489–1493; 10.1002/anie.201811675 30548141

[6] S. Kurumada , S. Takamori , M. Yamashita , Nat. Chem. 2020, 12, 36–39. 31767993 10.1038/s41557-019-0365-z

[7] K. Koshino , R. Kinjo , J. Am. Chem. Soc. 2020, 142, 9057–9062. 32321239 10.1021/jacs.0c03179

[8] R. J. Schwamm , M. P. Coles , M. S. Hill , M. F. Mahon , C. L. McMullin , N. A. Rajabi , A. S. S. Wilson , Angew. Chem. Int. Ed. 2020, 59, 3928–3932; 10.1002/anie.201914986 PMC7159655 31830364

[9] S. Grams , J. Eyselein , J. Langer , C. Färber , S. Harder , Angew. Chem. Int. Ed. 2020, 59, 15982–15986; 10.1002/anie.202006693 PMC7540686 32449816

[10] K. Sugita , M. Yamashita , Chem. Eur. J. 2020, 26, 4520–4523. 32052882 10.1002/chem.202000752

[11] K. Sugita , M. Yamashita , Organometallics 2020, 39, 2125–2129.

[12] J. Hicks , A. Mansikkamaki , P. Vasko , J. M. Goicoechea , S. Aldridge , Nat. Chem. 2019, 11, 237–241. 30664716 10.1038/s41557-018-0198-1

[13] A. Suzuki , X. Y. Guo , Z. Y. Lin , M. Yamashita , Chem. Sci. 2021, 12, 917–928. 10.1039/d0sc05478j PMC8179162 34163858

[14] M. Jansen , Chem. Soc. Rev. 2008, 37, 1826–1835. 18762832 10.1039/b708844m

[15] T. Andersen , H. K. Haugen , H. Hotop , J. Phys. Chem. Ref. Data 1999, 28, 1511–1533.

[16] D. S. Laitar , P. Muller , J. P. Sadighi , J. Am. Chem. Soc. 2005, 127, 17196–17197. 16332062 10.1021/ja0566679

[17] Y. Segawa , M. Yamashita , K. Nozaki , Angew. Chem. Int. Ed. 2007, 46, 6710–6713; 10.1002/anie.200702369 17665408

[18] T. Kajiwara , T. Terabayashi , M. Yamashita , K. Nozaki , Angew. Chem. Int. Ed. 2008, 47, 6606–6610; 10.1002/anie.200801728 18637644

[19] Y. Okuno , M. Yamashita , K. Nozaki , Angew. Chem. Int. Ed. 2011, 50, 920–923; 10.1002/anie.201005667 21246691

[20] K. Semba , M. Shinomiya , T. Fujihara , J. Terao , Y. Tsuji , Chem. Eur. J. 2013, 19, 7125–7132. 23576470 10.1002/chem.201300443

[21] Y. Okuno , M. Yamashita , K. Nozaki , Eur. J. Org. Chem. 2011, 3951–3958.

[22] C. Borner , C. Kleeberg , Eur. J. Inorg. Chem. 2014, 2486–2489.

[23] C. Kleeberg , C. Borner , Organometallics 2018, 37, 4136–4146.

[24] W. Drescher , C. Kleeberg , Inorg. Chem. 2019, 58, 8215–8229. 31148446 10.1021/acs.inorgchem.9b01041

[25] H. T. Zhao , Z. Y. Lin , T. B. Marder , J. Am. Chem. Soc. 2006, 128, 15637–15643. 17147372 10.1021/ja063671r

[26] H. T. Zhao , L. Dang , T. B. Marder , J. Am. Chem. Soc. 2008, 130, 5586–5594. 18373345 10.1021/ja710659y

[27] Z. H. Li , L. Zhang , M. Nishiura , Z. M. Hou , ACS Catal. 2019, 9, 4388–4393.

[28] N. N. Baughman , N. G. Akhmedov , J. L. Petersen , B. V. Popp , Organometallics 2021, 40, 23–37.

[29] S. P. Green , C. Jones , D. P. Mills , A. Stasch , Organometallics 2007, 26, 3424–3430.

[30] C. Jones , D. P. Mills , R. P. Rose , A. Stasch , W. Woodul , J. Organomet. Chem. 2010, 695, 2410–2417.

[31] C. Ganesamoorthy , J. Weßing , C. Kroll , R. W. Seidel , C. Gemel , R. A. Fischer , Angew. Chem. Int. Ed. 2014, 53, 7943–7947; 10.1002/anie.201402149 24962074

[32] J. Weßing , C. Ganesamoorthy , S. Kahlal , R. Marchal , C. Gemel , O. Cador , A. C. H. Da Silva , J. L. F. Da Silva , J.-Y. Saillard , R. A. Fischer , Angew. Chem. Int. Ed. 2018, 57, 14630–14634; 10.1002/anie.201806039 29981271

[33] K. L. Mears , C. R. Stennett , E. K. Taskinen , C. Knapp , C. J. Carmalt , H. M. Tuononen , P. P. Power , J. Am. Chem. Soc. 2020, 142, 19874–19878. 33170691 10.1021/jacs.0c10099

