# Supplementary material for: Ambiphilic Al−Cu Bonding
Source: Angew Chem Int Ed Engl. 2021 May 20;60(26):14390–3. doi: 10.1002/anie.202104658 (PMC8252794; doi:10.1002/anie.202104658)
Supplement: Supplementary file 1 — Supplementary [file ANIE-60-14390-s001.pdf]

Angewandte Chemie
Eine Zeitschrift der Gesellschaft Deutscher Chemiker

Supporting Information

Ambiphilic Al–Cu Bonding

Han-Ying Liu, Ryan J. Schwamm, Michael S. Hill, Mary F. Mahon, Claire L. McMullin,* and Nasir A. Rajabi*

anie_202104658_sm_miscellaneous_information.pdf

General information

Unless stated otherwise, all the experiments were conducted using standard Schlenk line and/or glovebox techniques under an inert atmosphere of argon. NMR spectra were recorded with an Agilent ProPulse spectrometer (^{1}H at 500 MHz, ^{13}C at 126 MHz). The spectra are referenced relative to residual protio solvent resonances. Elemental analyses were performed at Elemental Microanalysis Ltd., Okehampton, Devon, UK. Solvents were dried by passage through a commercially available solvent purification system and stored under argon in ampoules over 4 Å molecular sieves. C_6D_6 was purchased from Sigma-Aldrich, dried over a potassium mirror before distilling and storage over molecular sieves. [{SiN^{Dipp}}AlK]$_2$ (**XI**),[1] *N,N'*-diisopropyl-4,5-dimethyl-2-ylidene (NHC^{iPr}),[2] ^{Me2}CAAC,[3] ^{Me2}CAACCuCl[4] were prepared according to reported procedures. All other chemicals were purchased from Merck and used without further purification.

Synthetic Procedures

Synthesis of [{SiN^{Dipp}}Al-Cu{NHC^{iPr}}] (**1**)

A solution of *N,N'*-diisopropyl-4,5-dimethyl-2-ylidene (NHC^{iPr}, 0.166 g, 0.912 mmol) in toluene (10 mL) was added to a Schlenk flask containing CuCl (0.090 g, 0.912 mmol). After stirring for 2 hours at room temperature, a solution of [{SiN^{Dipp}}AlK]$_2$ (**XI**, 0.505 g, 0.455 mmol) in toluene (10 mL) was added to the stirring suspension and the resulting brown suspension was stirred at room temperature overnight. Removal of the volatile components *in vacuo*, followed by extraction into hexane and filtration gave a clear, colourless solution. Removal of the hexane solvent *in vacuo* gave a colourless powder of **1**. Colourless crystals of **1** were isolated from a saturated methylcyclohexane solution stored at –30 °C for 24 hours. Yield 0.61 g, 89 %. Anal Calc'd for $C_{41}H_{70}AlCuN_4Si_2$ (**1**.$(C_7H_{14})_{0.5}$, *814.12*): C, 65.52; H, 9.64; N, 6.87 %. Found: C, 65.49; H, 9.21; N, 6.51 %. ^{1}H NMR (500 MHz, 298K, Benzene-d_6): δ 7.14 (d, 4H, J = 7.6 Hz, *m*-C_6H_3), 7.03 (t, 2H, J = 7.6 Hz, *p*-C_6H_3), 4.21 (sept, 4H, J = 6.9 Hz, C*H*Me$_2$ on SiN^{Dipp}), 3.55 (sept, 2H, J = 6.8 Hz, C*H*Me$_2$ on NHC^{iPr}) 1.49 – 1.46 (m, 24H, CH*Me$_2$* on SiN^{Dipp}), 1.29 (s, 6H, NC*Me*), 1.27 (s, 4H, C*H$_2$*Si), 0.99 (d, 12H, J = 6.8 Hz, CH*Me$_2$* on NHC^{iPr}), 0.35 (s, 12H, Si*Me$_2$*). ^{13}C{^{1}H} NMR (126 MHz, 298K, Benzene-d$_6$) δ 175.9 (Cu*C* of NHC^{iPr}) 147.4 (*i*-*C*$_6$H$_3$), 147.1 (*o*-*C*$_6$H$_3$), 123.2 (*m*-*C*$_6$H$_3$), 122.8 (*p*-*C*$_6$H$_3$), 122.1 (N*C*Me) 49.7 (*C*HMe$_2$ on NHC^{iPr}), 28.3 (*C*HMe$_2$ on SiN^{Dipp}), 26.3, 24.5* (*C*HMe$_2$ on SiN^{Dipp} and NHC^{iPr}), 14.4 (*C*H$_2$Si), 8.6 (NC*Me*), 1.2 (Si*Me*$_2$). *two overlapping resonances

Figure S1. ^{1}H NMR Spectrum of **1** (500 MHz, C_6D_6); *silicone grease.

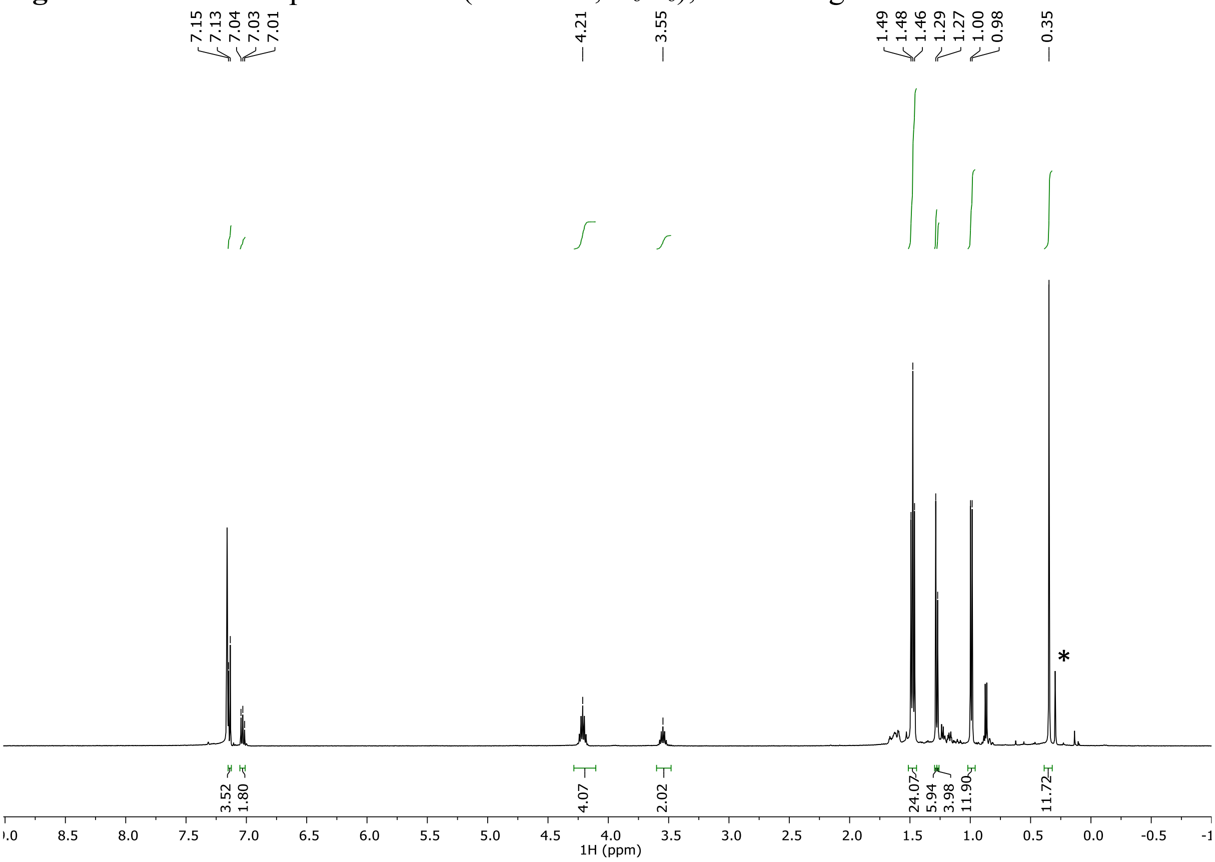

Figure S2. $^{13}C\{^1H\}$ NMR Spectrum of **1** (126 MHz, C_6D_6).

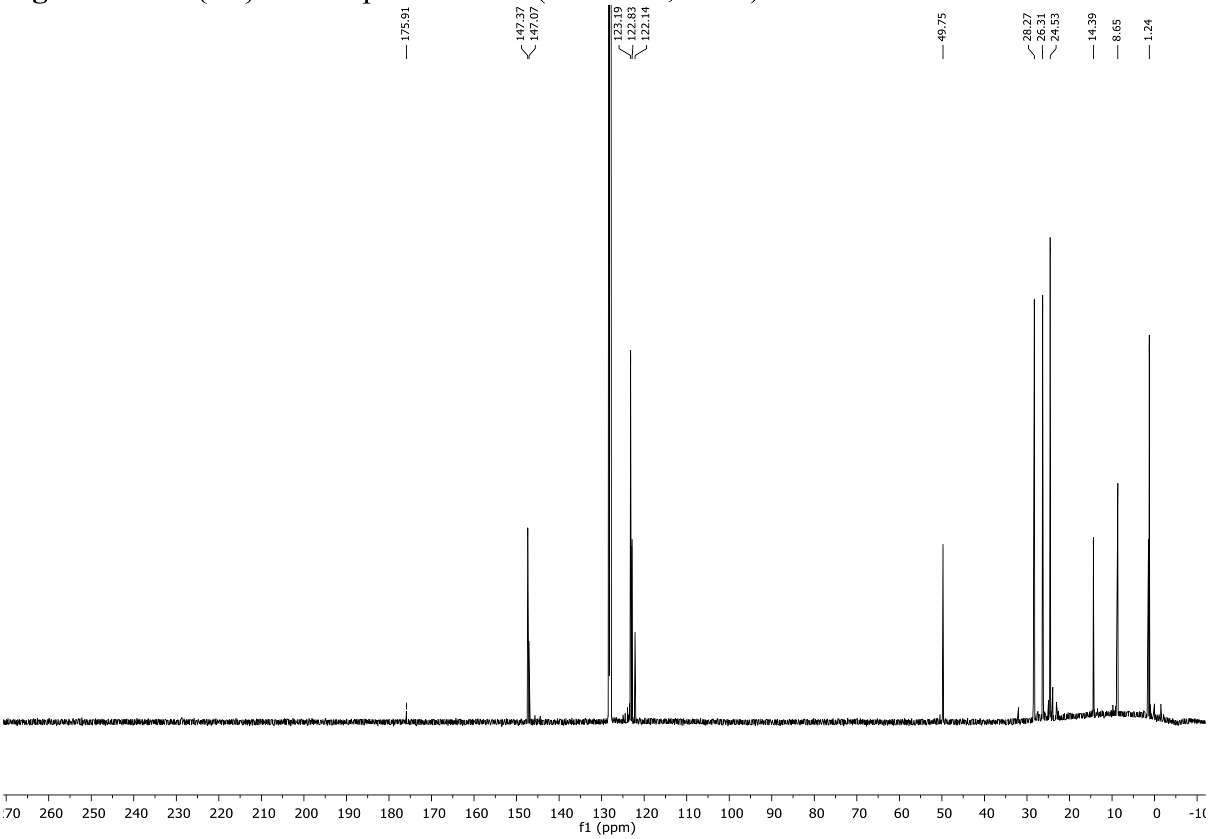

Figure S3. ^{1}H-^{1}H COSY NMR Spectrum of **1**.

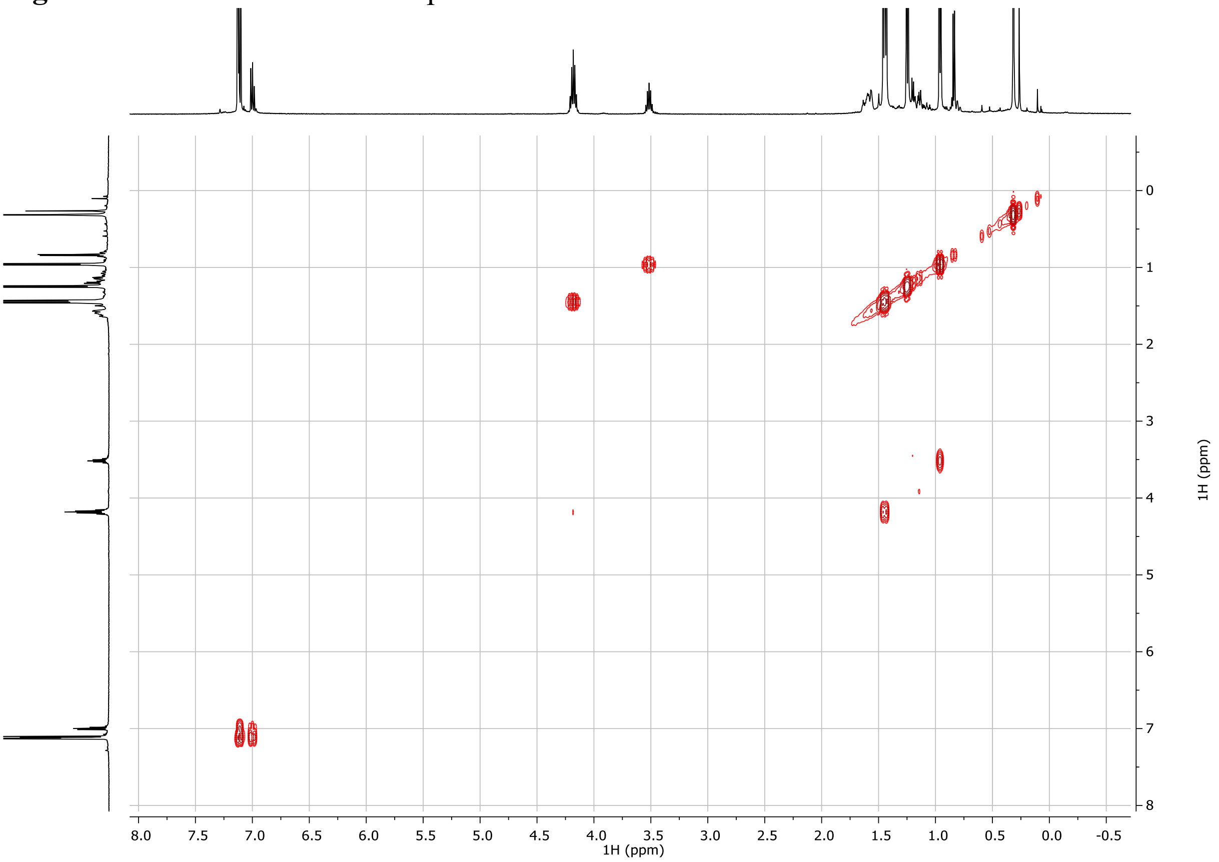

Figure S4. ^{1}H-^{13}C HSQC NMR Spectrum of **1**.

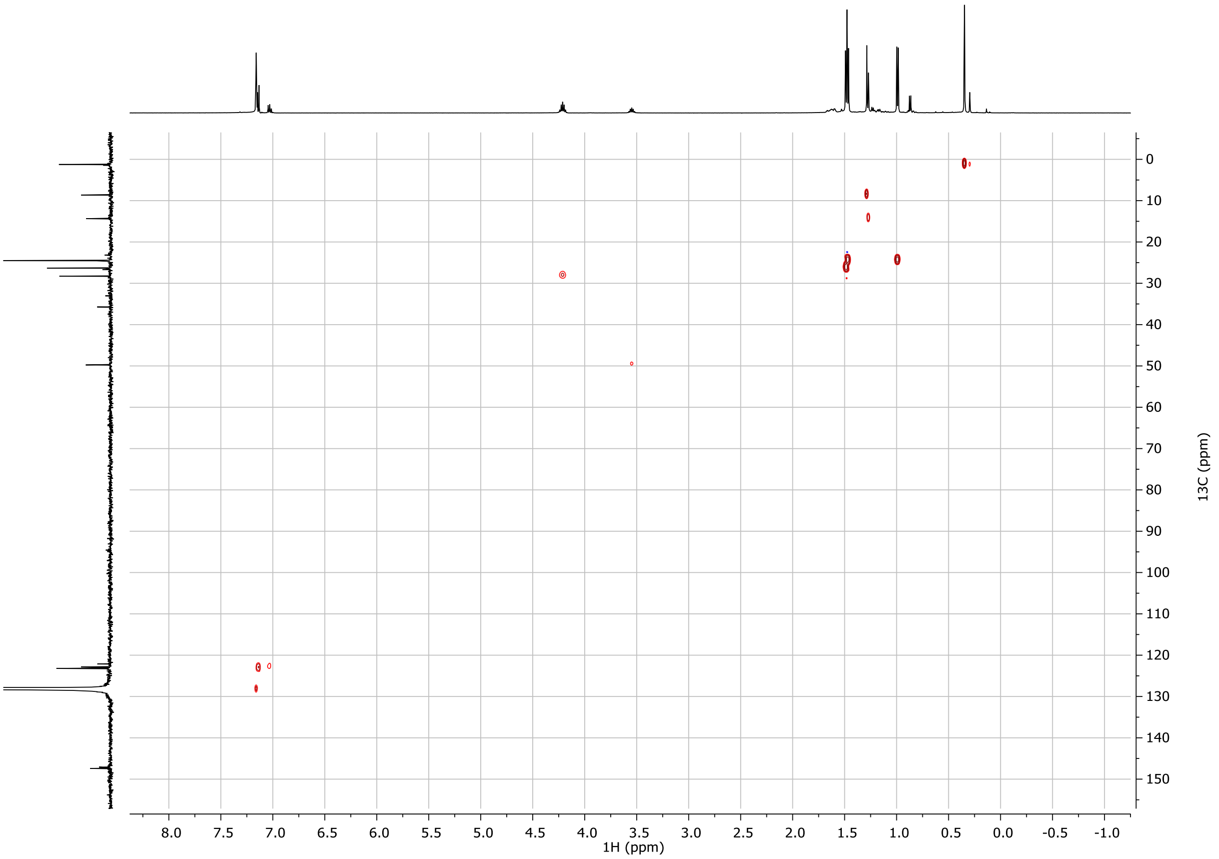

Figure S5. ^{1}H-^{13}C HMBC NMR Spectrum of **1**.

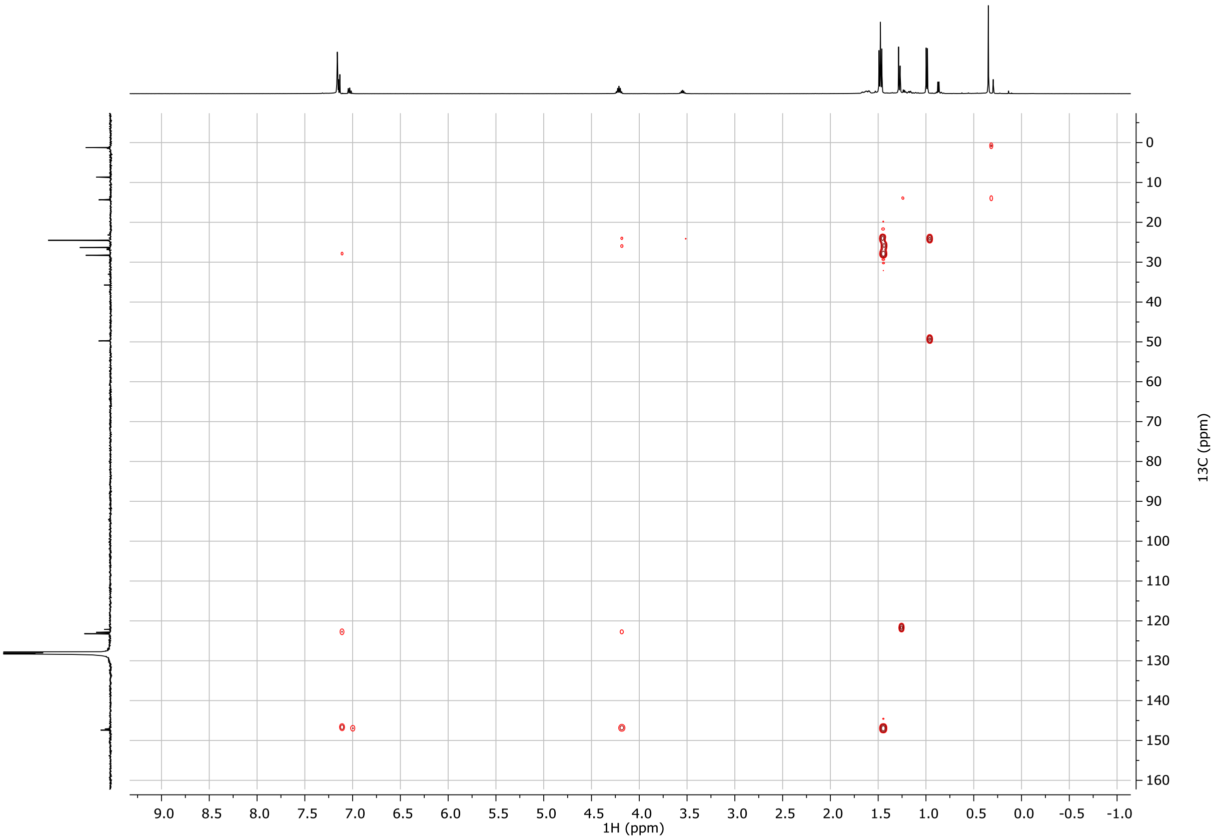

Synthesis of [{SiN^{Dipp}}Al-Cu{^{Me2}CAAC}] (**2**)

A solution of [{SiN^{Dipp}}AlK]$_2$ (**XI,** 0.560 g, 0.50 mmol) in hexane (20mL) was added dropwise into a stirring suspension of [{^{Me2}CAAC}CuCl] (0.384g, 1 mmol) in hexane (30mL) at room temperature. The mixture was stirred for 12 hours before filtering. The colourless filtrate was then collected, and all volatiles were removed *in vacuo* yielding **2** as a white solid. Yield 0.688 g, 79%. Colourless crystals suitable for X-ray diffraction analysis were obtained by slow evaporation of a hexane solution at room temperature. No meaningful result was obtained for elemental analysis after multiple attempts. ^{1}H NMR (500 MHz, Benzene-d_6) δ 7.14 – 7.09 (m, 4H, *m*-C$_6$*H*$_3$ on SiN^{Dipp}), 7.09 – 7.03 (m, 3H, *p*-C$_6$*H*$_3$ on SiN^{Dipp} and ^{Me2}CAAC), 6.89 (d, 2H, J = 7.8 Hz, *m*-C$_6$*H*$_3$ on ^{Me2}CAAC), 4.05 (sept, 4H, J = 6.9 Hz, C*H*Me$_2$ on SiN^{Dipp}), 2.40 (sept, 2H, J = 6.8 Hz, C*H*Me$_2$ on ^{Me2}CAAC), 1.43, 1.26 (d, 12H, J = 6.9 Hz, CH*Me*$_2$ on SiN^{Dipp}), 1.19 (s, 2H, CMe$_2$C*H*$_2$CMe$_2$), 1.14 (s, 4H, SiC*H*$_2$), 1.02 (d, 6H, J = 6.8 Hz, CH*Me*$_2$ on ^{Me2}CAAC), 0.85 (d, 6H, J = 6.8 Hz, CH*Me*$_2$ on ^{Me2}CAAC), 0.81 (s, 6H, C*Me*$_2$), 0.68 (s, 6H, NC*Me*$_2$CH$_2$ on ^{Me2}CAAC), 0.27 (s, 12H, Si*Me*$_2$). ^{13}C{^{1}H} NMR (126 MHz, Benzene-d$_6$) δ 254.2 (Cu*C* on ^{Me2}CAAC), 147.0 (*i-C*$_6$H$_3$ on SiN^{Dipp}), 146.8 (*o-C*$_6$H$_3$ on SiN^{Dipp}), 144.9 (*i-C*$_6$H$_3$ on ^{Me2}CAAC), 134.5 (*o-C*$_6$H$_3$ on ^{Me2}CAAC), 129.4 (*p-C*$_6$H$_3$ on ^{Me2}CAAC), 124.6 (*m-C*$_6$H$_3$ on ^{Me2}CAAC), 123.4 (*m-C*$_6$H$_3$ on SiN^{Dipp}), 122.7 (*p-C*$_6$H$_3$ on SiN^{Dipp}), 80.6 (N*C*Me$_2$CH$_2$), 55.7 (*C*Me$_2$CH$_2$CMe$_2$), 50.1 (CMe$_2$*C*H$_2$CMe$_2$), 29.1 (*C*HMe$_2$ on ^{Me2}CAAC), 28.9 (CH*Me*$_2$ on ^{Me2}CAAC), 28.7 (NC*Me*$_2$CH$_2$), 28.2 (*C*HMe$_2$ on SiN^{Dipp}), 27.3 (CH*Me*$_2$ on ^{Me2}CAAC), 26.4 (CH*Me*$_2$ on SiN^{Dipp}), 24.5 (CH*Me*$_2$ on SiN^{Dipp}), 22.5 (CH*Me*$_2$ on ^{Me2}CAAC), 14.6 (Si*C*H$_2$), 1.6 (Si*Me*$_2$).

Figure S6. ^{1}H NMR Spectrum of **2** (500 MHz, 298K, C_6D_6).

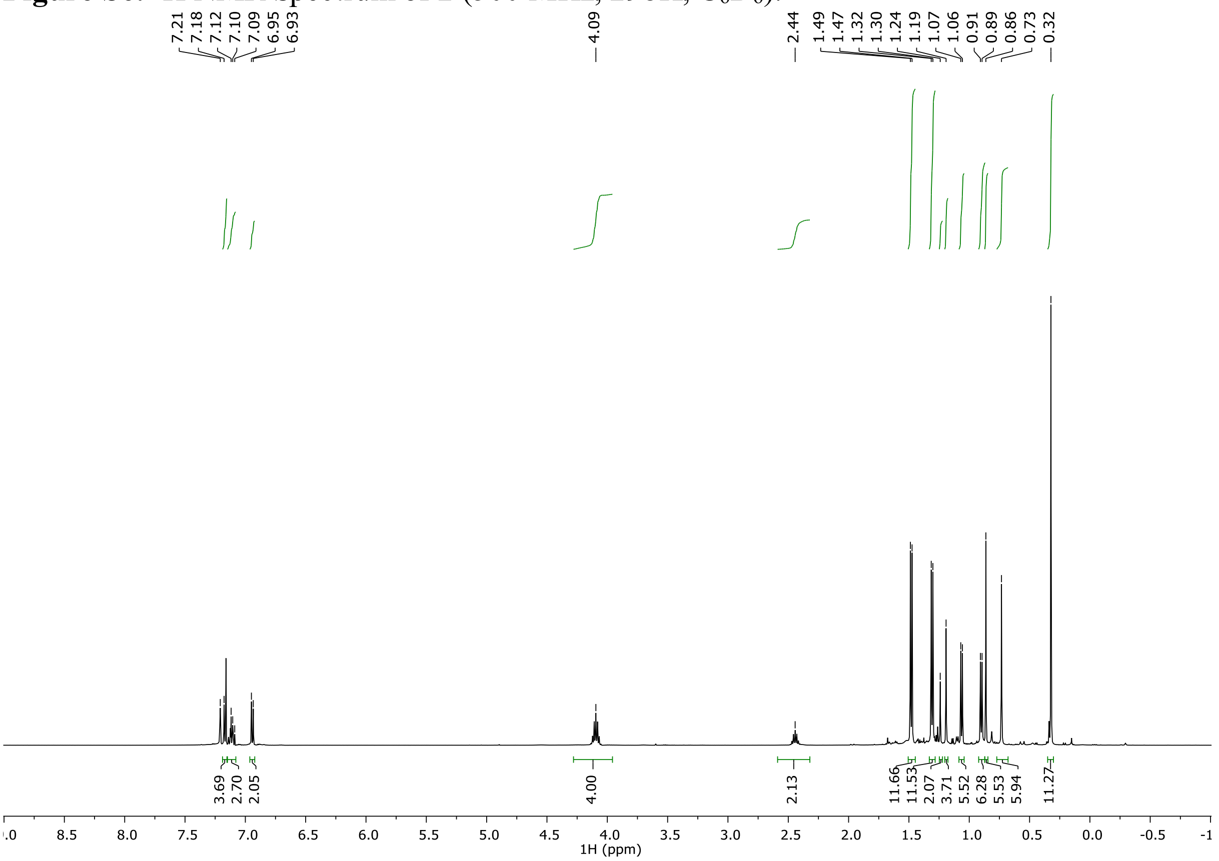

Figure S7. $^{13}C\{^{1}H\}$ NMR Spectrum of **2** (500 MHz, 298K, C_6D_6).

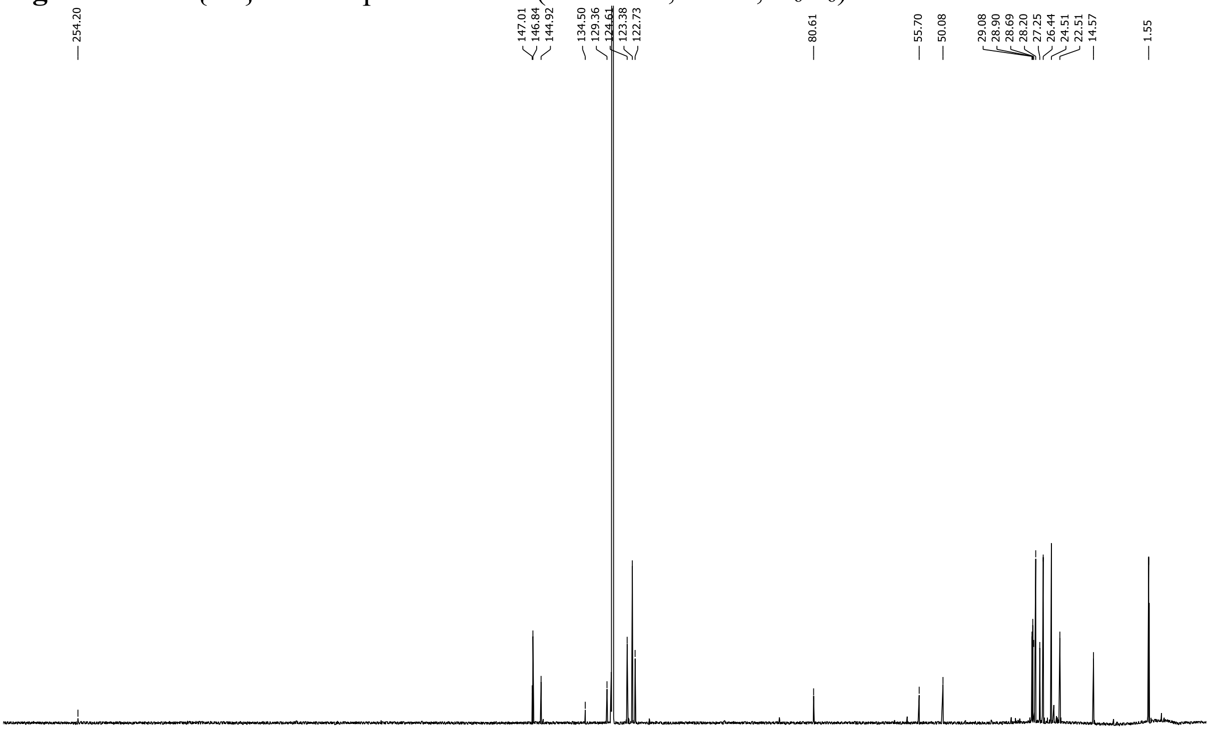

Figure S8. ^{1}H-^{1}H COSY NMR Spectrum of **2**.

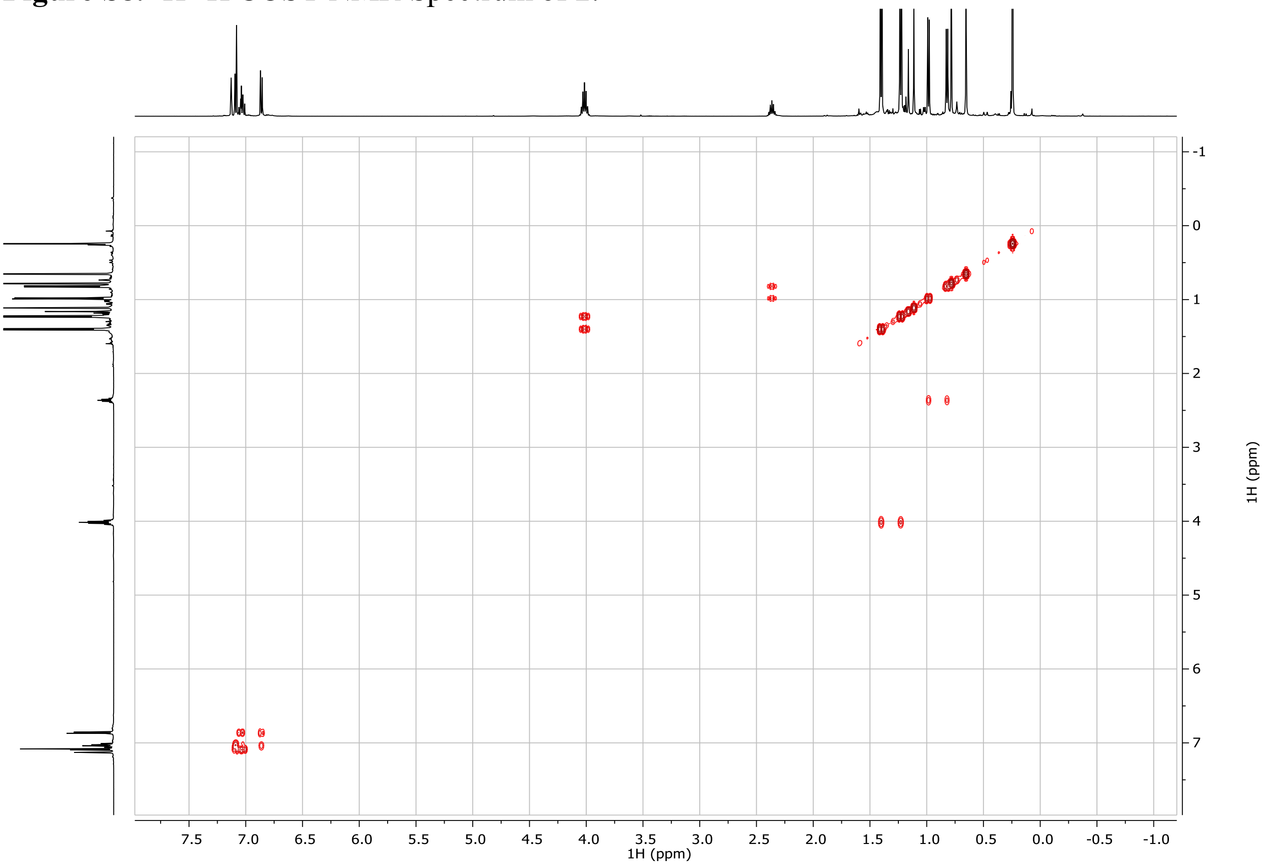

Figure S9. ^{1}H-^{13}C HSQC NMR Spectrum of **2**.

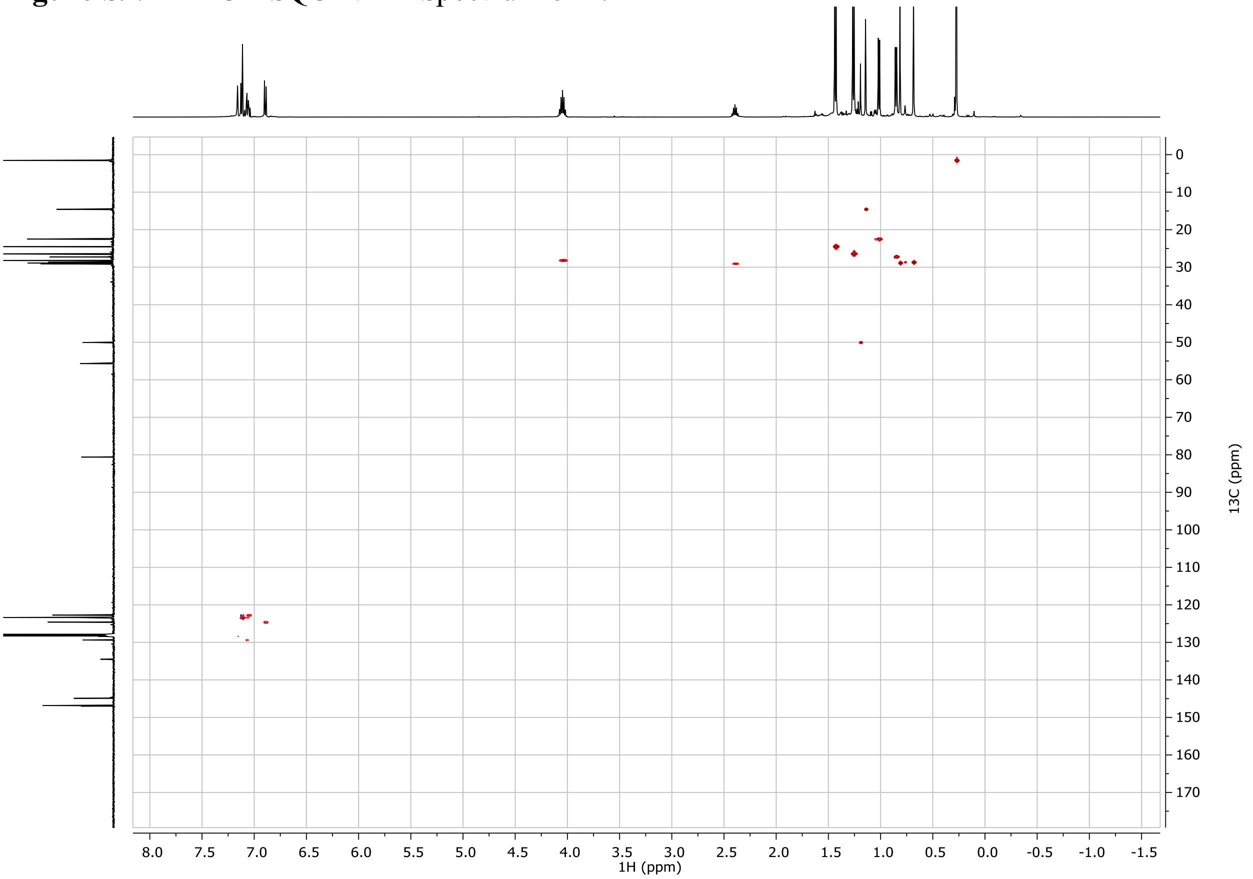

Figure S10. ^1H-^{13}C HMBC NMR Spectrum of **2**.

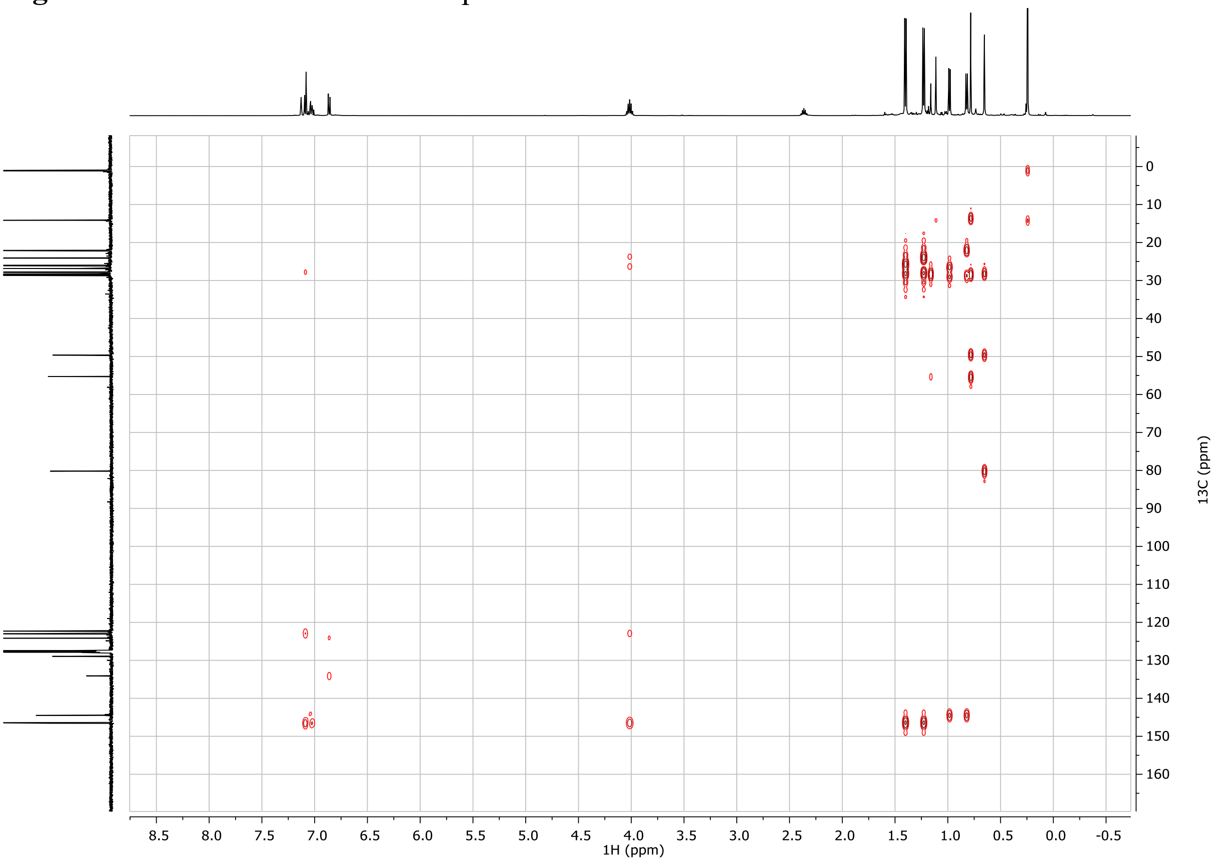

Synthesis of [{SiN^{Dipp}}Al-C(NiPr)$_2$-Cu{NHC^{iPr}}] (**3**)

In a J Young's NMR tube, [{SiN^{Dipp}}Al-Cu{NHC^{iPr}}] (**1**, 25 mg, 0.033 mmol) was dissolved in 0.4 mL of C_6D_6. *N,N'*-di-isopropylcarbodiimide (5 µL, 0.033mmol) was then added *via* micropipette. No significant change was observed in the ^{1}H NMR spectrum within one hour of the mixing of the starting materials. The reaction mixture was then left at room temperature overnight, forming compound **3** in quantitative yield (determined by ^{1}H NMR). The benzene solution was then put under reduced pressure to remove all volatiles giving **3** as colourless solid. Crystals suitable for X-ray diffraction analysis were obtained by slow evaporation of a hexane solution at room temperature. Yield 20 mg, 69%. Anal Calc'd for $C_{51}H_{91}AlCuN_6Si_2$ (**3**.$(C_6H_{14})_{0.5}$, *935.04*): C, 65.51; H, 9.81; N, 8.99 %. Found: C, 65.66; H, 9.79; N, 8.66 %. ^{1}H NMR (500 MHz, Benzene-*d*$_6$) δ 7.26 – 7.22 (m, 4H, *m*-C_6H_3 on SiN^{Dipp}), 7.15 – 7.11 (m, 2H, *p*- C_6H_3 on SiN^{Dipp}), 4.46 – 4.17 (m, 7H, C*H*Me$_2$ on SiN^{Dipp} and NC*H*Me$_2$), 3.36-3.27 (m, 1H, NC*H*Me$_2$ of carbodiimide), 1.60 (d, 6H, J = 6.8 Hz, CH*Me*$_2$ on SiN^{Dipp}), 1.57-1.53 (m, 6H, CH*Me*$_2$, CH*Me*$_2$ on SiN^{Dipp}) 1.53-1.48 (m, 12H, CH*Me*$_2$, CH*Me*$_2$ on SiN^{Dipp}), 1.39-1.33 (m, 4H, SiC*H*$_2$) 1.35 (s, 6H, NC*Me*), 1.29-1.19 (m, 6H, NCH*Me*$_2$ on carbodiimide) , 1.13 (d, J = 7.0 Hz, 12H, NCH*Me*$_2$ on NHC), 1.03-0.89 (m, 6H, NCH*Me*$_2$ of carbodiimide), 0.54 - 0.25 (br, 12H, Si*Me*$_2$). ^{13}C{^{1}H} NMR (126 MHz, Benzene-d$_6$) δ 146.8 (*i*-C_6H$_3$),146.6 (*o*-C_6H$_3$), 124.0 (N*C*Me$_2$), 123.7 (*m*-C_6H$_3$), 122.7 (*p*-C_6H$_3$), 57.6 (N*C*HMe$_2$ of carbodiimide), 52.2 (N*C*HMe$_2$ on NHC^{iPr}), 44.9 (N*C*HMe$_2$ of carbodiimide), 32.0 (CH*Me*$_2$), 28.1 (CH*Me*$_2$), 28.0 (*C*HMe$_2$), 27.6 (*C*HMe$_2$), 26.5, 26.3, 25.9 (CH*Me*$_2$), 23.2 (NCH*Me*$_2$ on NHC^{iPr}), 15.1 (Si*C*H$_2$), 9.0 (NC*Me*), 1.98 (Si*Me*$_2$); Cu-*C$_{carbene}$*, Al-*C*N$_2$ not observed.

Figure S11. ^{1}H NMR Spectrum of **3** (500 MHz, 298K, C_6D_6); *silicone grease, $^{\#}$ hexane.

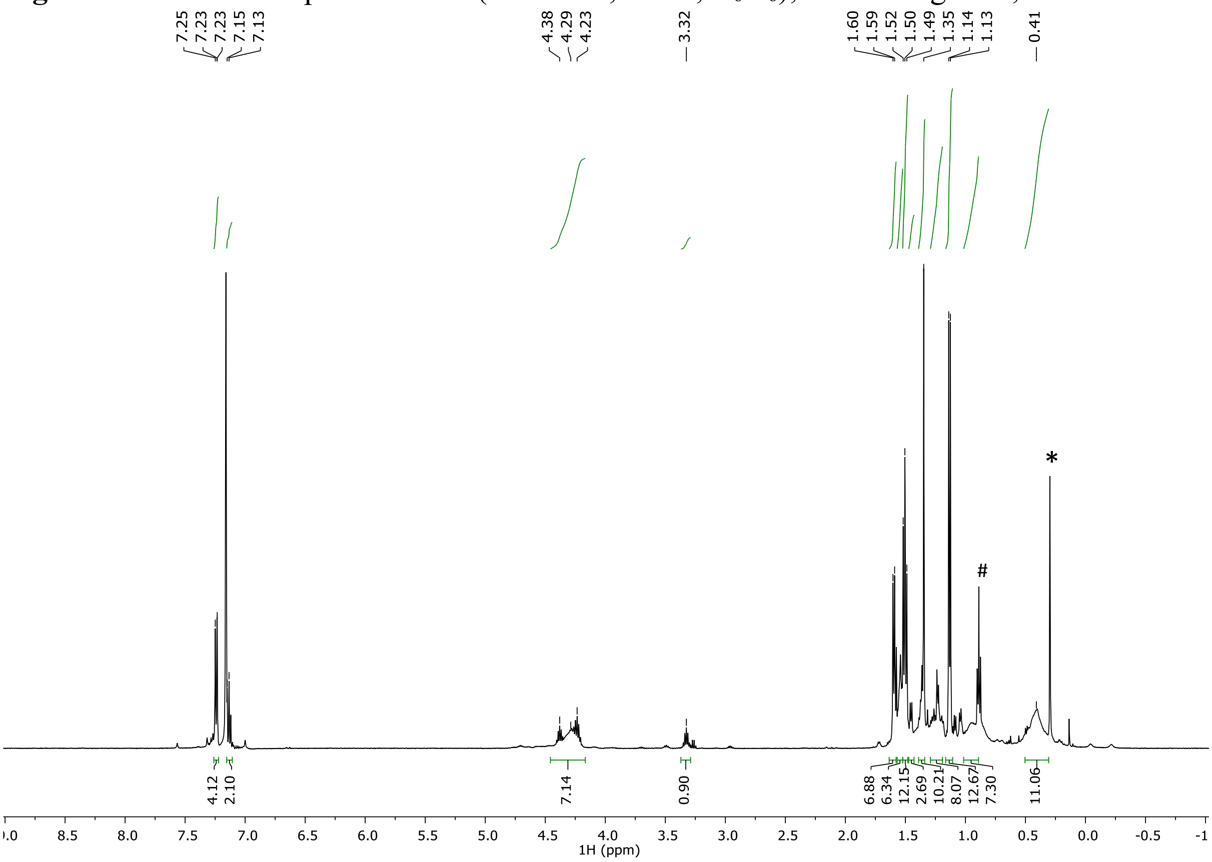

Figure S12. $^{13}C\{^{1}H\}$ NMR Spectrum of **3** (500 MHz, 298K, C_6D_6); *silicone grease, $^{\#}$ hexane.

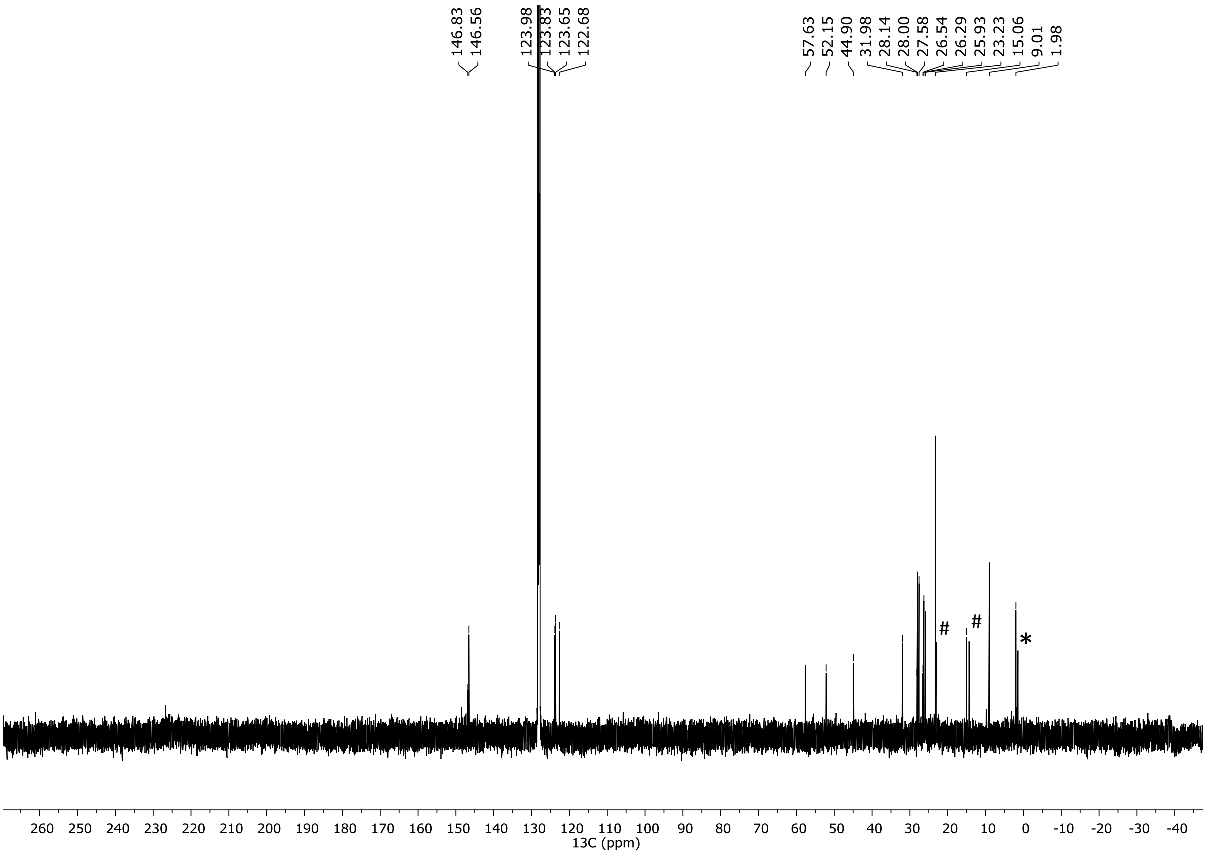

Figure S13. ^{1}H-^{1}H COSY NMR Spectrum of **3**.

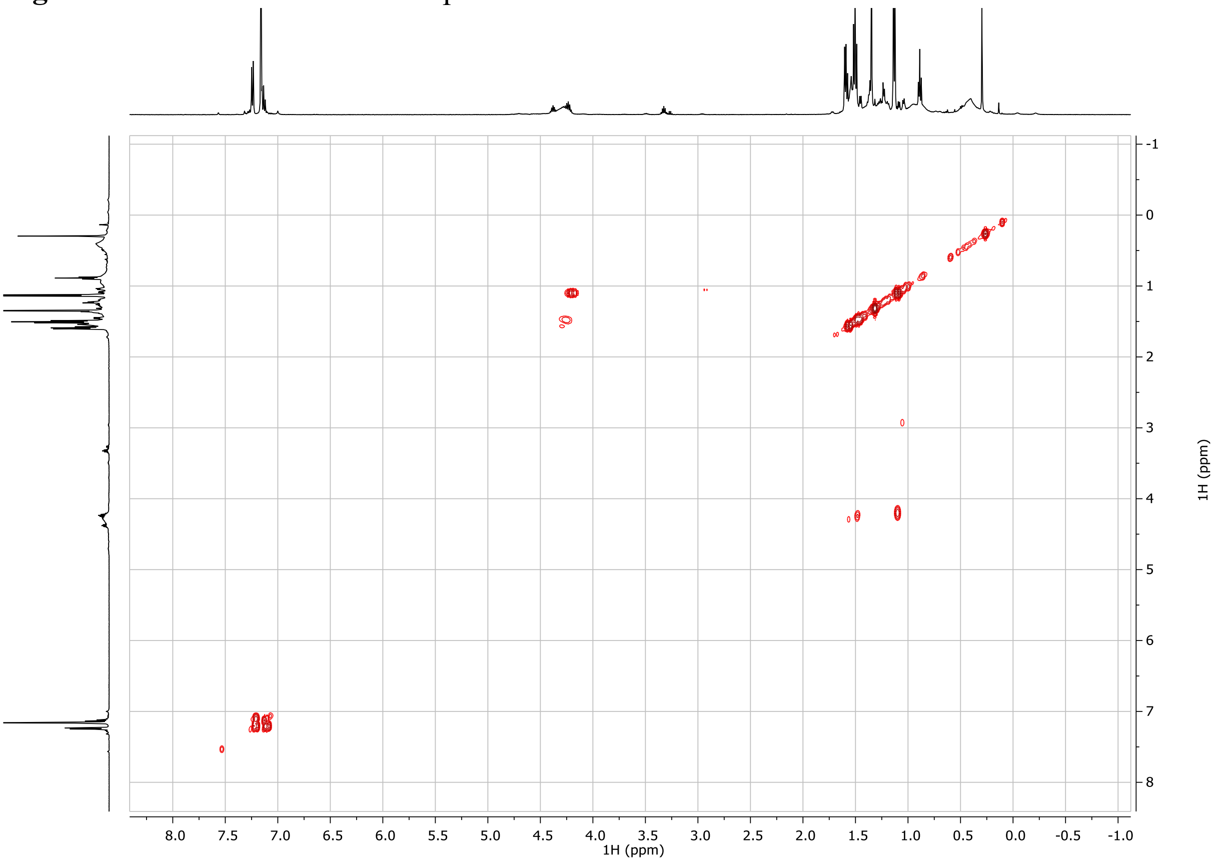

Figure S14. ^{1}H-^{13}C HSQC NMR Spectrum of **3**.

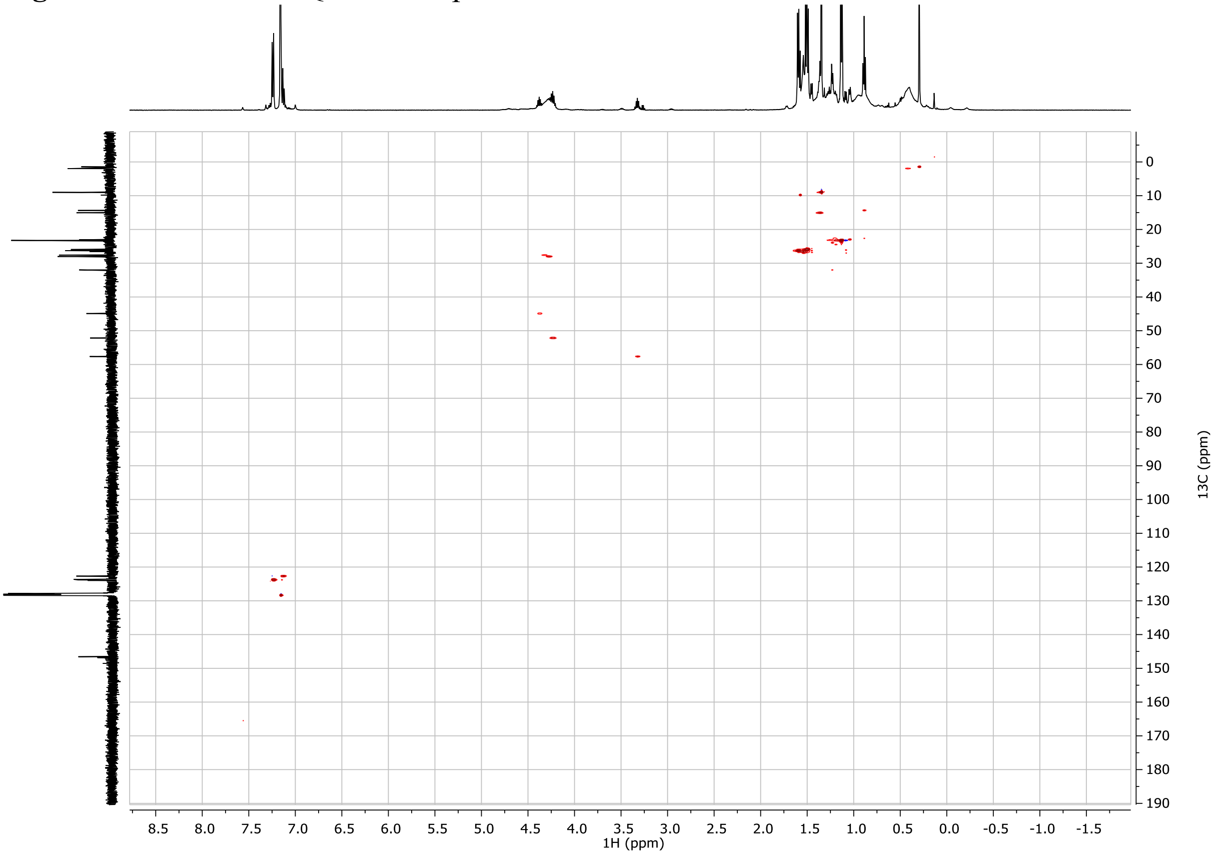

Figure S15. ^{1}H-^{13}C HMBC NMR Spectrum of **3**.

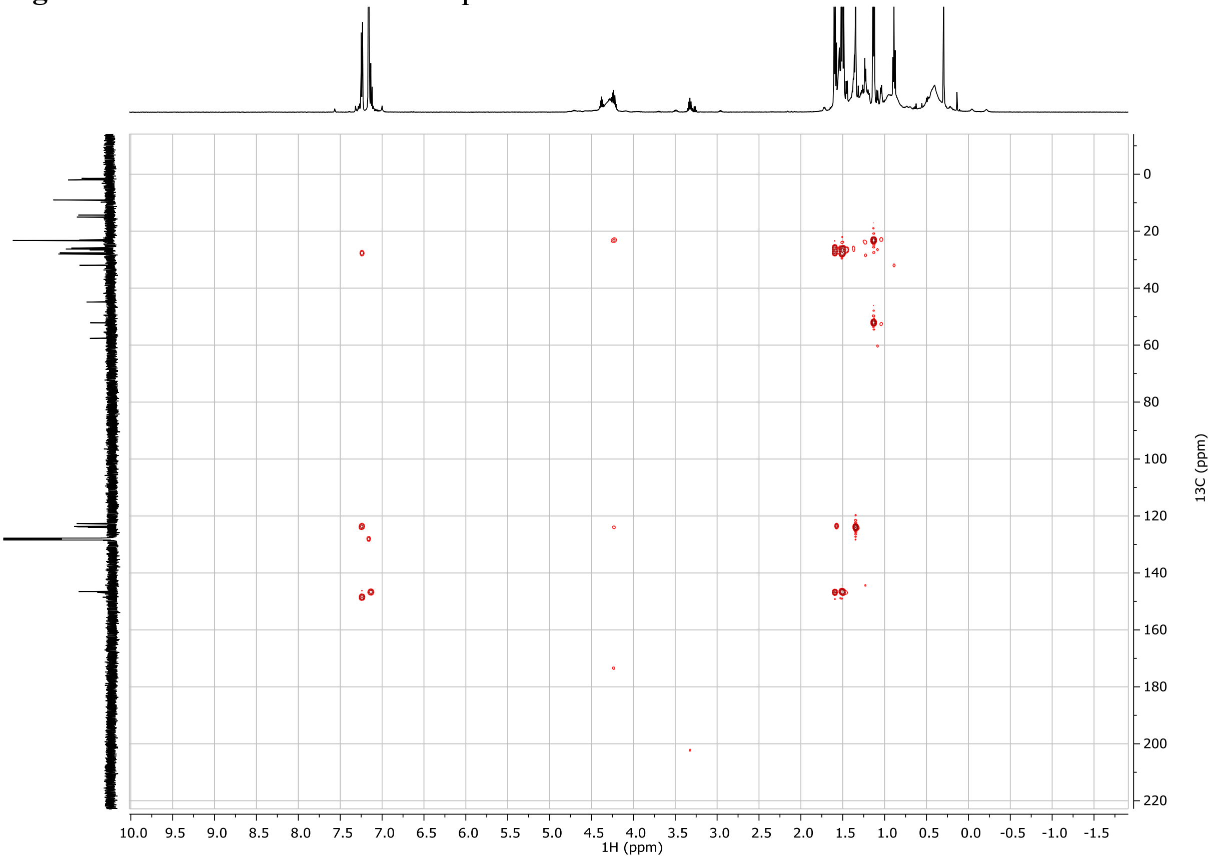

Synthesis of [{SiN^{Dipp}}Al-C(NiPr)$_2$-Cu{^{Me2}CAAC}] (**4**)

In a J Young's NMR tube, [{SiN^{Dipp}}Al-Cu{^{Me2}CAAC}] (**2**, 43.5mg, 0.05mmol) was dissolved in 0.4mL of C_6D_6, *N,N'*-di-isopropylcarbodiimide (7.8µL, 0.05mmol) was then added *via* a micropipette. No significant change was observed within one hour of the mixing of the starting materials by ^{1}H NMR. The reaction mixture was then left at room temperature overnight, cleanly forming the inserted product. The benzene solution was then put under reduced pressure to remove all volatiles and giving **4** as a colourless solid. Crystals suitable for X-ray diffraction analysis were obtained by slow evaporation of a hexane solution at room temperature. Yield 36 mg, 72%. Anal Calc'd for $C_{57}H_{95}AlCuN_5Si_2$ (**4**, *997.12*): C, 68.66; H, 9.60; N, 7.02 %. Found: C, 68.68; H, 9.42; N, 6.82 %. ^{1}H NMR (500 MHz, Benzene-*d*$_6$) δ 7.24-7.18 (m, 4H, *m*-C_6H_3 on SiN^{Dipp}), 7.14 – 7.08 (m, 2H, *p*-C_6H_3 on SiN^{Dipp}), 6.98 (t, 1H, J = 7.7 Hz, *p*-C_6H_3 on ^{Me2}CAAC), 6.84 (d, 2H, J = 7.7 Hz, *m*-C_6H_3 on ^{Me2}CAAC), 4.47 – 4.13 (m, 4H, C*H*Me$_2$ on SiN^{Dipp}), 3.57 – 3.19 (m, 2H, NC*H*Me$_2$), 2.54-2.43 (m, 2H, C*H*Me$_2$ on ^{Me2}CAAC), 1.51 – 1.37 (m, 30H, CH*Me*$_2$ on SiN^{Dipp} and NCH*Me*$_2$), 1.27 (s, 2H, CMe$_2$C*H*$_2$CMe$_2$ on ^{Me2}CAAC), 1.17 (d, J = 6.6 Hz, 2H, CH*Me*$_2$ on ^{Me2}CAAC), 1.10 (s, 4H, SiC*H*$_2$), 1.05 (d, 6H, J = 6.9 Hz, NCH*Me*$_2$), 1.02 (d, 6H, J = 6.6 Hz, CH*Me*$_2$ on ^{Me2}CAAC), 0.78 (br s, 12H, C*Me*$_2$ on ^{Me2}CAAC), 0.52-0.21 (br, 12H, Si*Me*$_2$). ^{13}C{^{1}H} NMR (126 MHz, Benzene-*d*$_6$) δ 253.8 (Cu*C*), 220.9 (Cu*C*N$_2$), 149.7 (*i*-*C*$_6$H$_3$ on SiN^{Dipp}), 146.8 (*o*-*C*$_6$H$_3$ on SiN^{Dipp}), 144.8 (*i*-*C*$_6$H$_3$ on ^{Me2}CAAC), 135.2 (*o*-*C*$_6$H$_3$ on ^{Me2}CAAC), 129.9 (*p*-*C*$_6$H$_3$ on ^{Me2}CAAC), 125.1 (*m*-*C*$_6$H$_3$ on ^{Me2}CAAC) 124.1 (*m*-*C*$_6$H$_3$ on SiN^{Dipp}), 122.5 (*p*-*C*$_6$H$_3$ on SiN^{Dipp}), 81.0 (N*C*Me$_2$CH$_2$), 54.9 (*C*Me$_2$CH$_2$), 51.9 (N*C*HMe$_2$), 49.9 (CMe$_2$*C*H$_2$CMe$_2$), 29.2 (*C*HMe$_2$), 29.0 (C*Me*$_2$ on CAAC), 28.1, 27.7, 26.9, 26.7, 26.7, 26.6, 26.5, 24.8, 23.0 (CH*Me*$_2$), 15.2 (SiCH$_2$), 4.1 (Si*Me*$_2$).

Figure S16. ^{1}H NMR Spectrum of **4** (500 MHz, 298K, C_6D_6); *silicone grease.

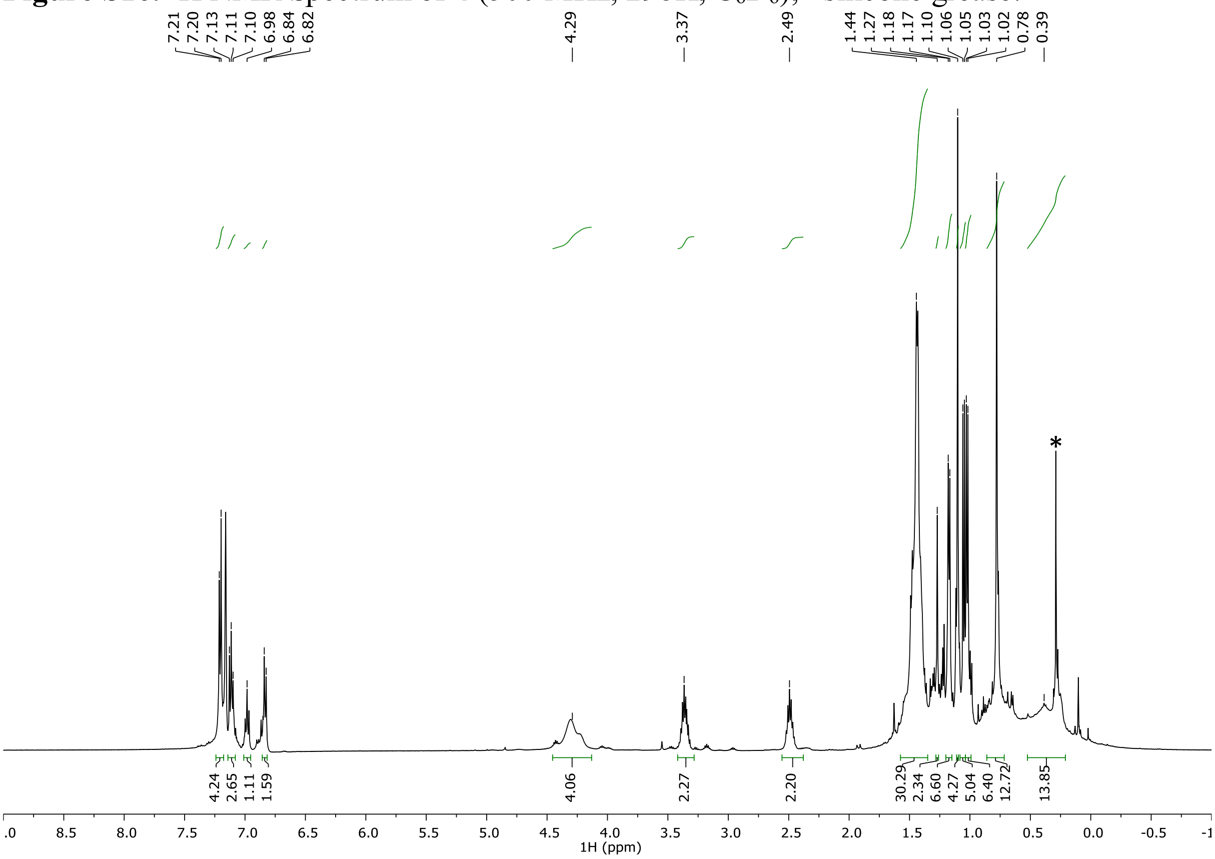

Figure S17. ^{13}C{^{1}H} NMR Spectrum of **4** (500 MHz, 298K, C_6D_6); *silicone grease.

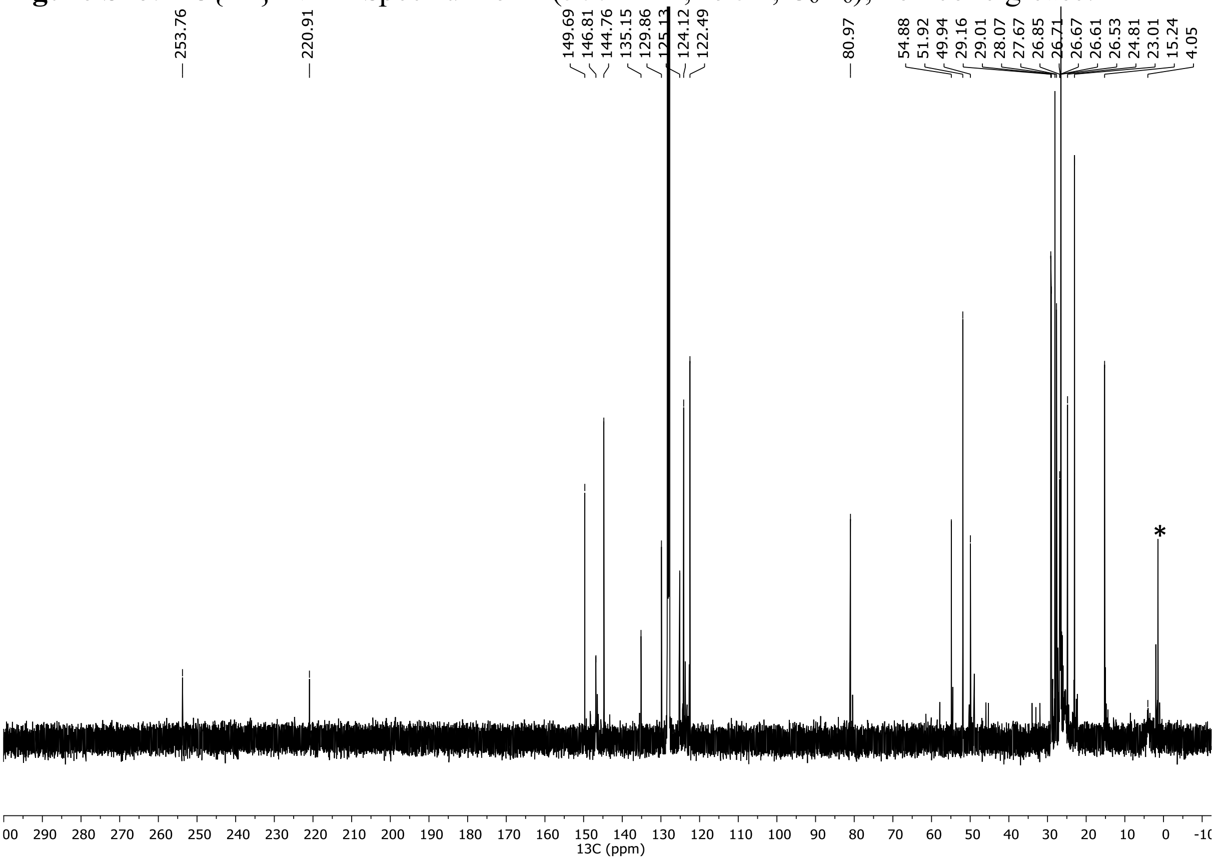

Figure S18. ^{1}H-^{1}H COSY NMR Spectrum of **4**.

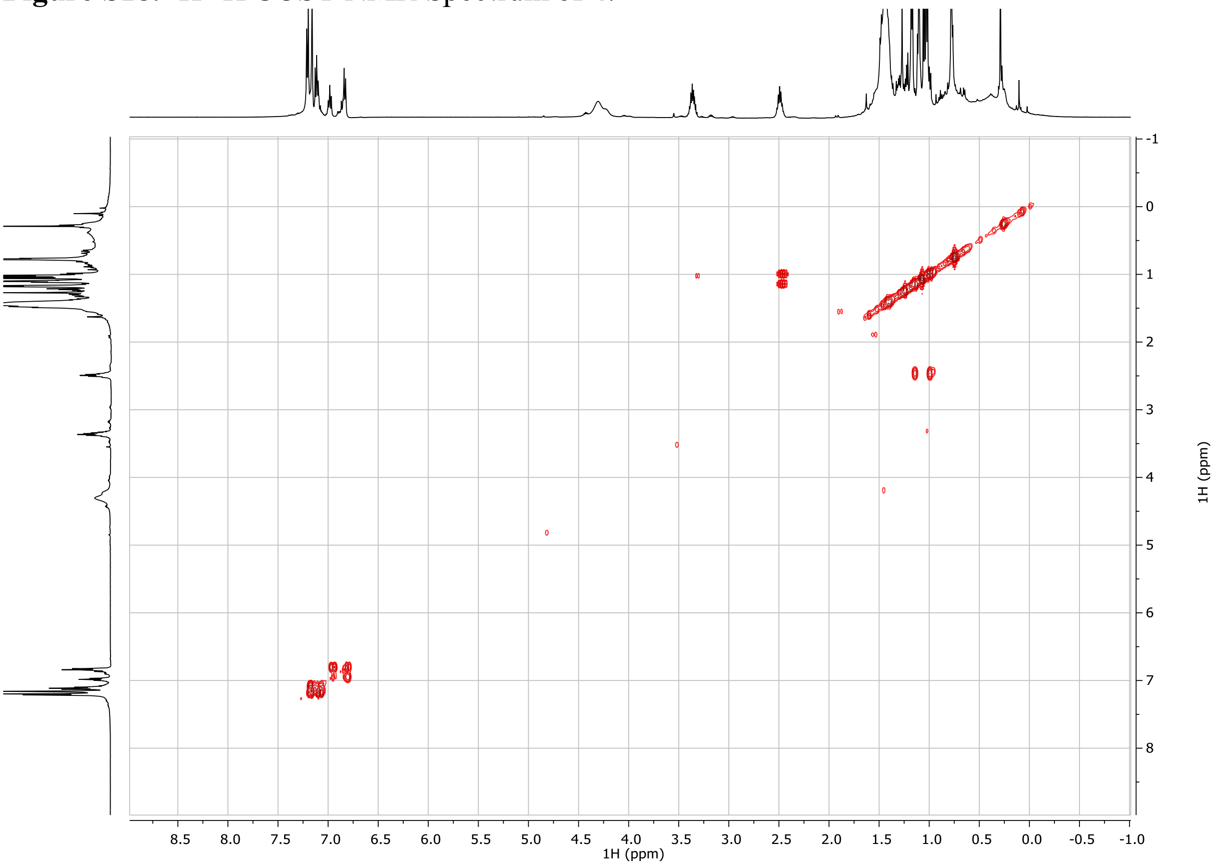

Figure S19. ^{1}H-^{13}C HSQC NMR Spectrum of **4**.

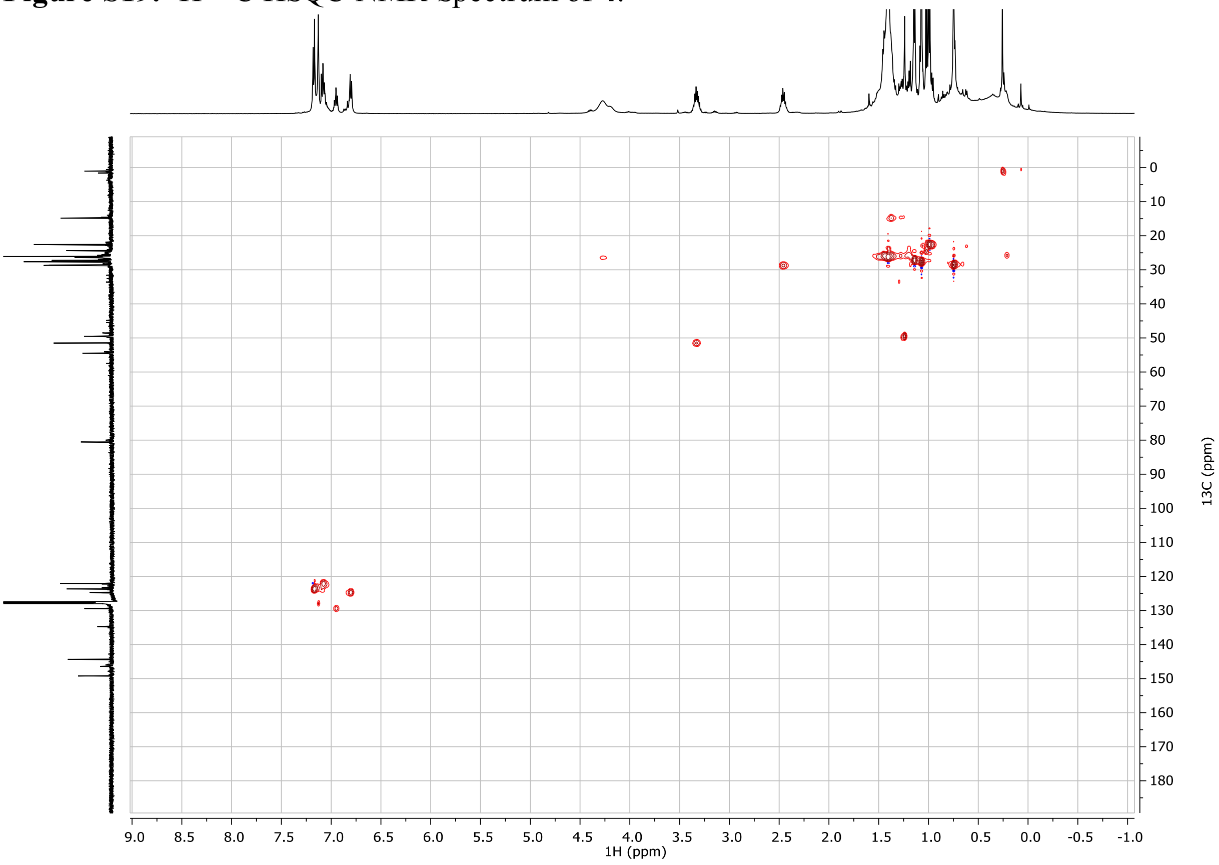

Figure S20. ^{1}H-^{13}C HMBC NMR Spectrum of **4**.

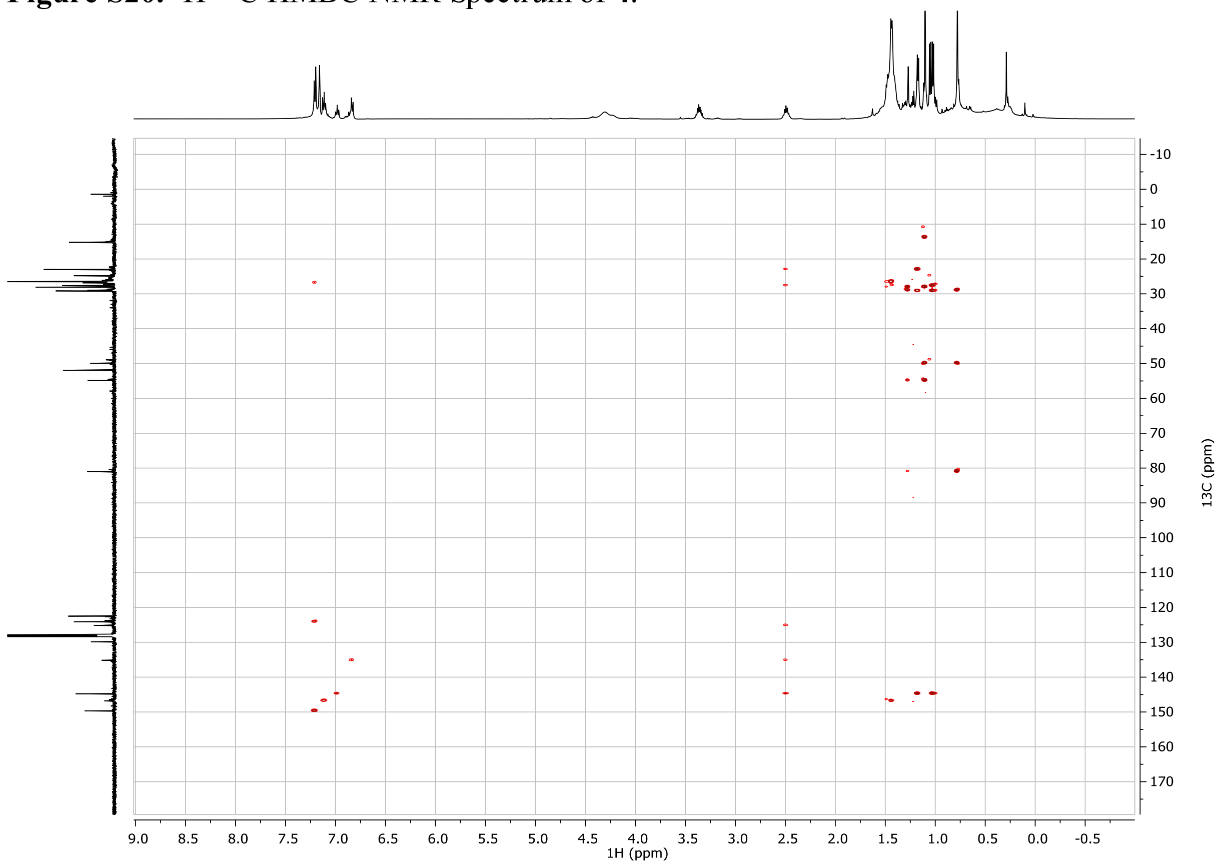

Synthesis of [{SiN^{Dipp}}Al-O_2C-Cu{NHC^{iPr}}] (**5**)

[{SiN^{Dipp}}Al-Cu{NHC^{iPr}}] (**1**, 25mg, 0.033mmol) was dissolved in 0.4 mL of C_6D_6 in a J Young's NMR tube. The solution was then degassed by three cycles of freeze-pump-thaw before the tube was charged with 2 atm of $^{13}CO_2$. Full Conversion of the starting material was determined by ^{1}H and ^{13}C NMR spectra within 30 minutes of the addition of the CO_2 to the solution. The benzene solution was then put under reduced pressure to remove all volatiles and giving **5** as colourless waxy solid. Yield 22 mg, 82%. No meaningful result was obtained for elemental analysis after multiple attempts. ^{1}H NMR (500 MHz, Benzene-*d*$_6$) δ 7.18 (d, 4H, *J* = 7.6 Hz, *m*-C_6H_3), 7.07 (t, 2H, *J* = 7.6 Hz, *p*-C_6H_3), 4.23 (sept, 4H, *J* = 6.8 Hz, C*H*Me_2 on SiN^{Dipp}), 3.51 (sept, 2H, *J* = 6.8 Hz, NC*H*Me_2 on NHC^{iPr}), 1.60 (d, 12H, *J* = 6.8 Hz, CH*Me*$_2$ on SiN^{Dipp}), 1.48 (d, 12H, *J* = 6.8 Hz, CH*Me*$_2$ on SiN^{Dipp}), 1.30 (s, 4H, SiC*H*$_2$), 1.21 (s, 6H, NC*Me*), 1.03 (d, 12H, *J* = 6.8 Hz, NCH*Me*$_2$), 0.40 (s, 12H, Si*Me*$_2$). ^{13}C{^{1}H} NMR (126 MHz, Benzene-*d*$_6$) δ 236.2 (Cu*C*O_2), 166.7 (Cu*C* of NHC^{iPr})146.9 (*o-C*$_6$H$_3$), 145.3 (*i-C*$_6$H$_3$), 123.5 (*m-C*$_6$H$_3$ on SiN^{Dipp}), 123.2 (N*C*Me on NHC^{iPr}), 123.1 (*p-C*$_6$H$_3$ on SiN^{Dipp}), 49.8 (N*C*HMe_2), 28.0, 25.6, 25.5 (*C*H*Me*$_2$ on SiN^{Dipp}), 24.6 (NCH*Me*$_2$), 14.5 (Si*C*H$_2$), 8.4 (NC*Me*), 0.6 (Si*Me*$_2$); *Resonance at 124.8 corresponds to residual $^{13}CO_2$.

Figure S21. ^{1}H NMR Spectrum of **5** (500 MHz, 298K, C_6D_6).

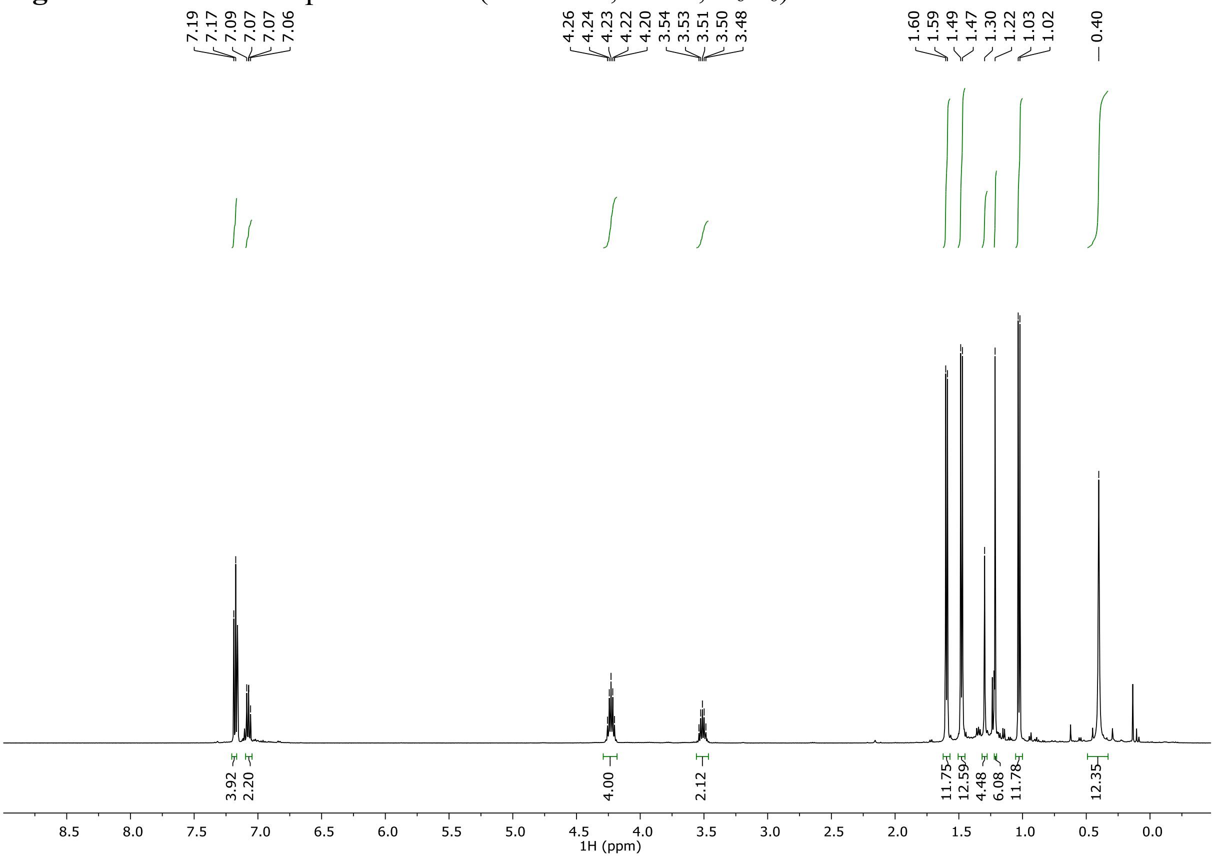

Figure S22. $^{13}C\{^{1}H\}$ NMR Spectrum of **5** (500 MHz, 298K, C_6D_6); *residual $^{13}CO_2$.

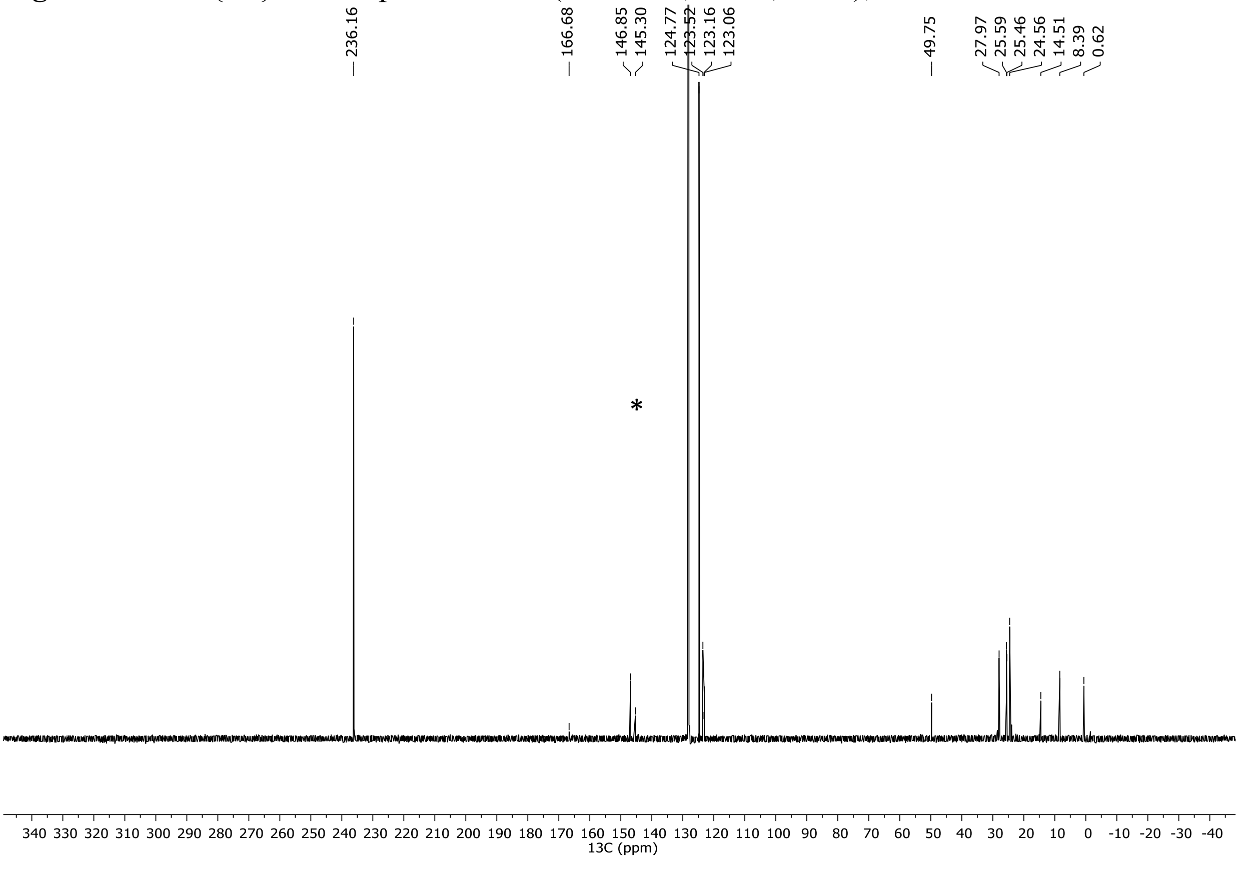

Figure S23. ^{1}H-^{1}H COSY NMR Spectrum of **5**.

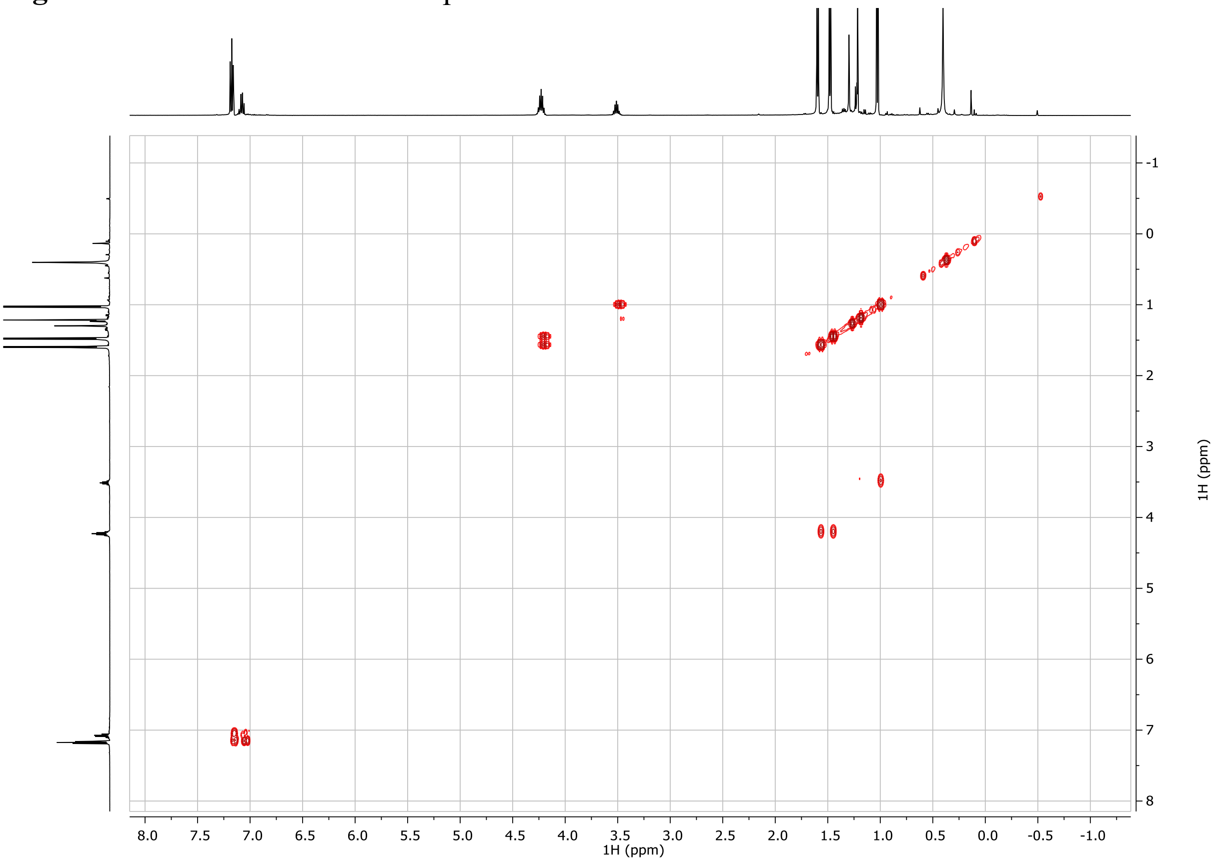

Figure S24. ^{1}H-^{13}C HSQC NMR Spectrum of **5**.

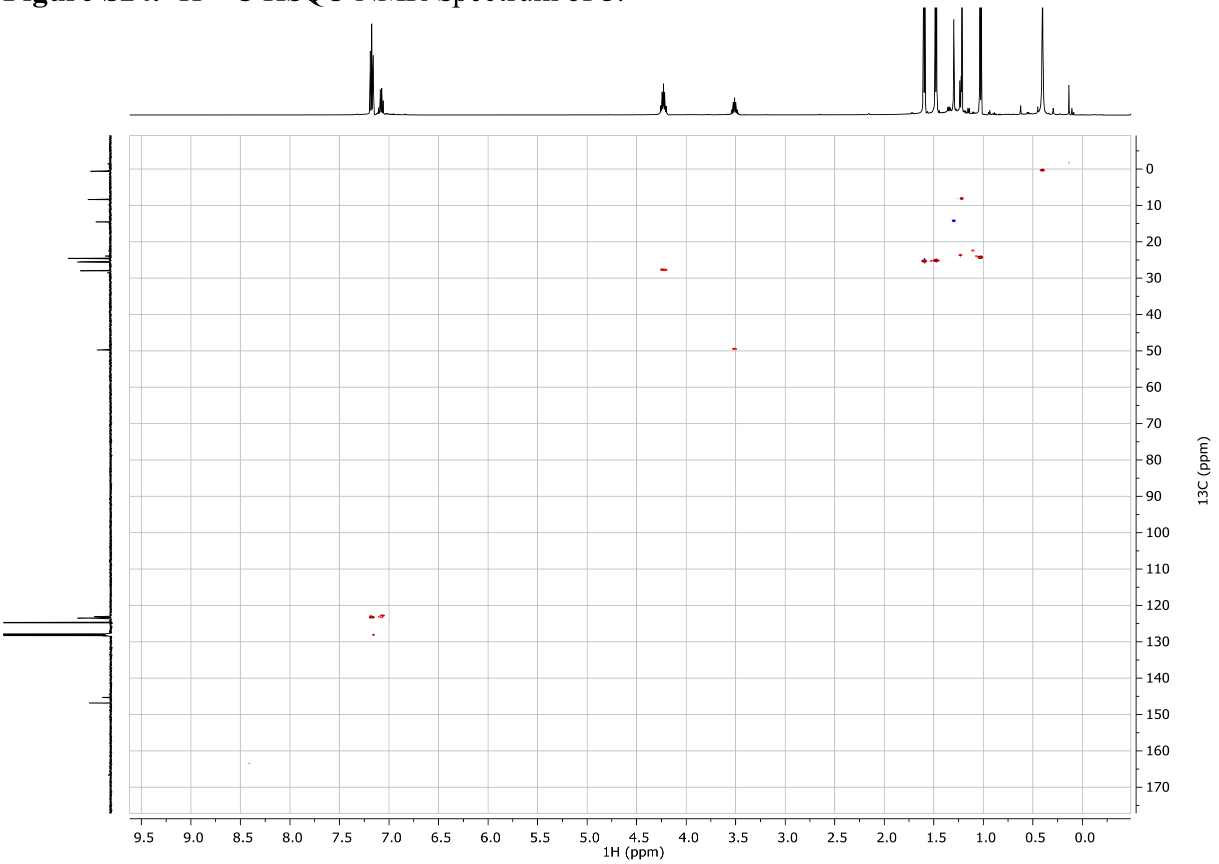

Figure S25. ^{1}H-^{13}C HMBC NMR Spectrum of **5**.

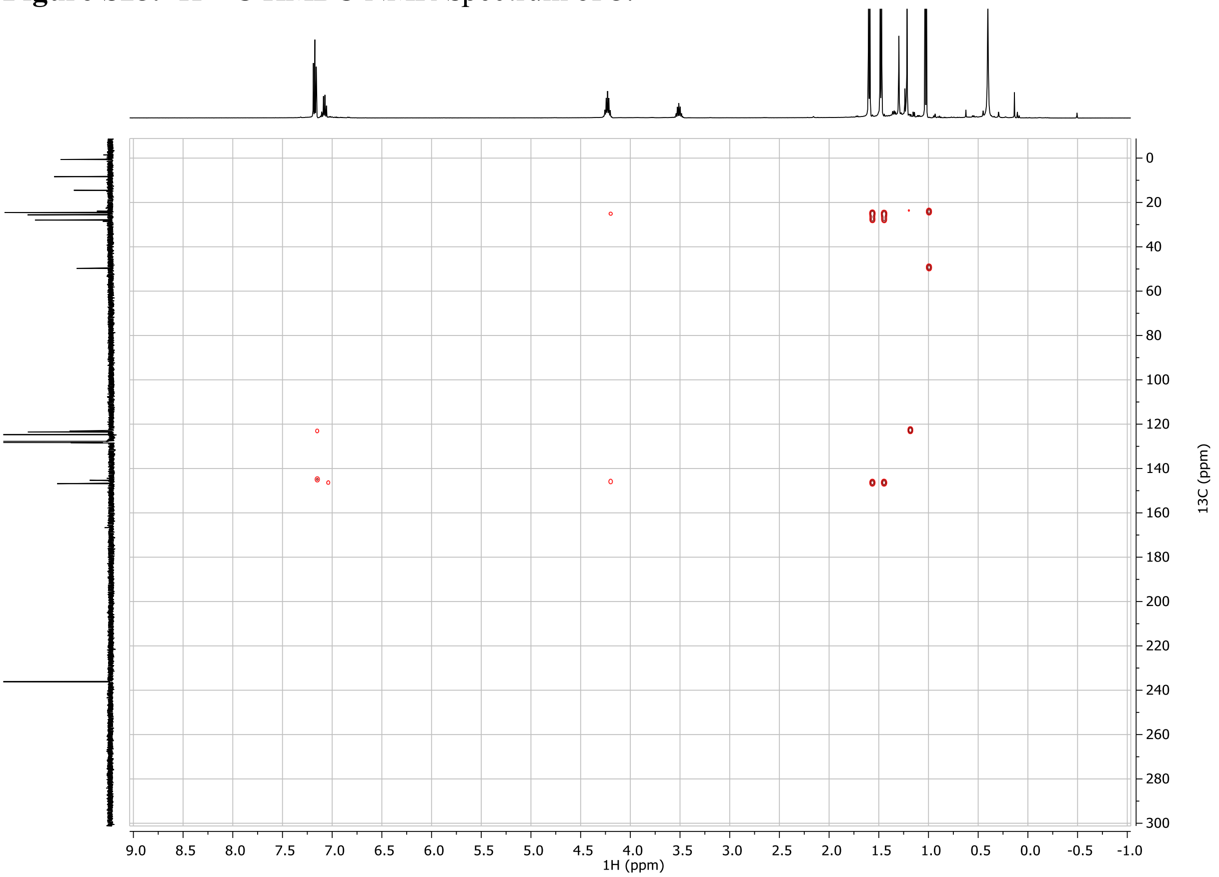

Synthesis of [{SiN^{Dipp}}Al-O_2C-Cu{^{Me2}CAAC}] (**6**)

[{SiN^{Dipp}}Al-Cu{^{Me2}CAAC}] (**2**, 43.5mg, 0.05mmol) was dissolved in 0.4mL of C_6D_6 inside a J Young's NMR tube. The solution was then degassed by three cycles of freeze-pump-thaw before the tube was charged with 2 atm of $^{13}CO_2$. Full Conversion of the starting material was determined by ^{1}H and ^{13}C NMR spectra within 30 minutes of the addition of the CO_2 to the solution. The benzene solution was then put under reduced pressure to remove all volatiles and giving **6** as colourless solid. Crystals suitable for X-ray diffraction analysis were obtained by slow evaporation of a hexane solution at room temperature. Yield 31 mg, 68%. No meaningful result was obtained for elemental analysis after multiple attempts. ^{1}H NMR (500 MHz, Benzene-d_6) δ 7.14 – 7.08 (m, 5H, *m*-C_6H_3 on SiN^{Dipp} and *p*-C_6H_3 on ^{Me2}CAAC), 7.07 – 7.02 (m, 2H, *p*-C_6H_3 on SiN^{Dipp}), 6.92 (d, 2H, J = 7.8 Hz, *m*-C_6H_3 on ^{Me2}CAAC), 4.05 (p, 4H, J = 6.9 Hz, C*H*Me_2 on SiN^{Dipp}), 2.33 (sept, 2H, J = 6.8 Hz, C*H*Me_2 on ^{Me2}CAAC), 1.42 (d, 12H, J = 6.9 Hz, CHMe_2 on SiN^{Dipp}), 1.35 (d, 12H, J = 6.9 Hz, CHMe_2 on SiN^{Dipp}), 1.23 (s, 4H, SiCH_2), 1.17 (s, 2H, $CMe_2CH_2CMe_2$), 1.03 (d, 6H, J = 6.8 Hz, CHMe_2 on ^{Me2}CAAC), 0.99 (d, 6H, J = 6.8 Hz, CHMe_2 on ^{Me2}CAAC), 0.84 (s, 6H, $CMe_2CH_2CMe_2$), 0.66 (s, 6H, NCMe_2CH_2), 0.33 (s, 1[2H, SiMe_2). ^{13}C{^{1}H} NMR (126 MHz, Benzene-d_6) δ 251.6 (Cu*C*), 234.9 (CuCO_2), 146.6 (*i*-C_6H_3 on SiN^{Dipp}), 145.0, 145.0 (*o*-C_6H_3 on SiN^{Dipp} and *i*-C_6H_3 on ^{Me2}CAAC), 133.7 (*o*-C_6H_3 on ^{Me2}CAAC), 130.1 (*p*-C_6H_3 on ^{Me2}CAAC), 124.9 (*m*-C_6H_3 on ^{Me2}CAAC), 123.4 (*m*-C_6H_3 on SiN^{Dipp}), 122.97 (*p*-C_6H_3 on SiN^{Dipp}), 81.0 (NCMe_2CH_2), 53.9 ($CMe_2CH_2CMe_2$), 49.1 ($CMe_2CH_2CMe_2$, 29.1 (CHMe_2 on ^{Me2}CAAC), 28.5 (NCMe_2CH_2), 27.8 (CHMe_2 on SiN^{Dipp}), 27.7 (C$Me_2CH_2CMe_2$), 27.1 (CHMe_2 on ^{Me2}CAAC), 25.5 (CH*Me*2 on SiN^{Dipp}), 22.5 (CHMe_2 on ^{Me2}CAAC), 14.5 (SiCH_2), 0.5 (SiMe_2); Resonance at 124.8 corresponds to residual $^{13}CO_2$, only impurities at 165-175ppm, no correlation with proton observed in ^{1}H-^{13}C HSQC, HMBC, plausibly ^{13}C labelled minor impurities.

Figure S26. ^{1}H NMR Spectrum of **6** (500 MHz, C_6D_6); *silicone grease.

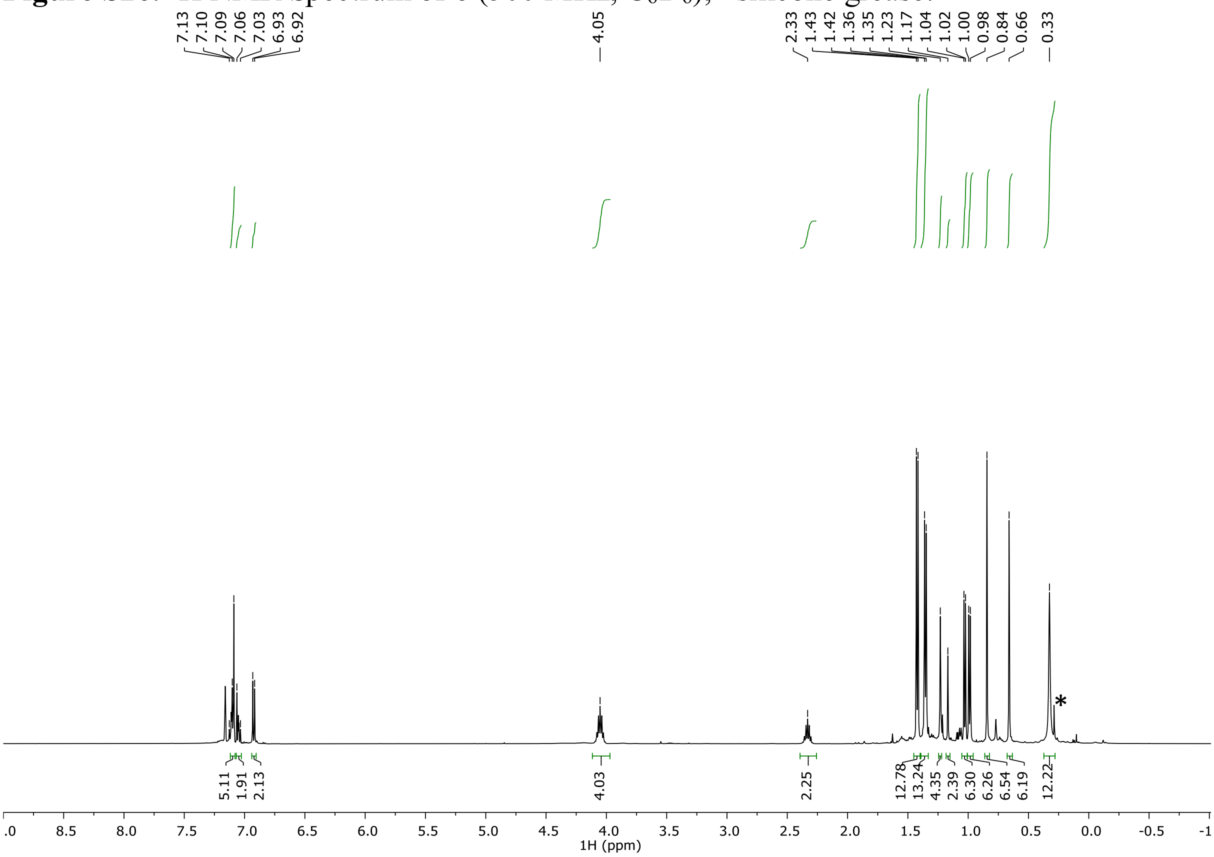

Figure S27. $^{13}C\{^{1}H\}$ NMR Spectrum of **6** (500 MHz, C_6D_6); *silicone grease, #residual $^{13}CO_2$.

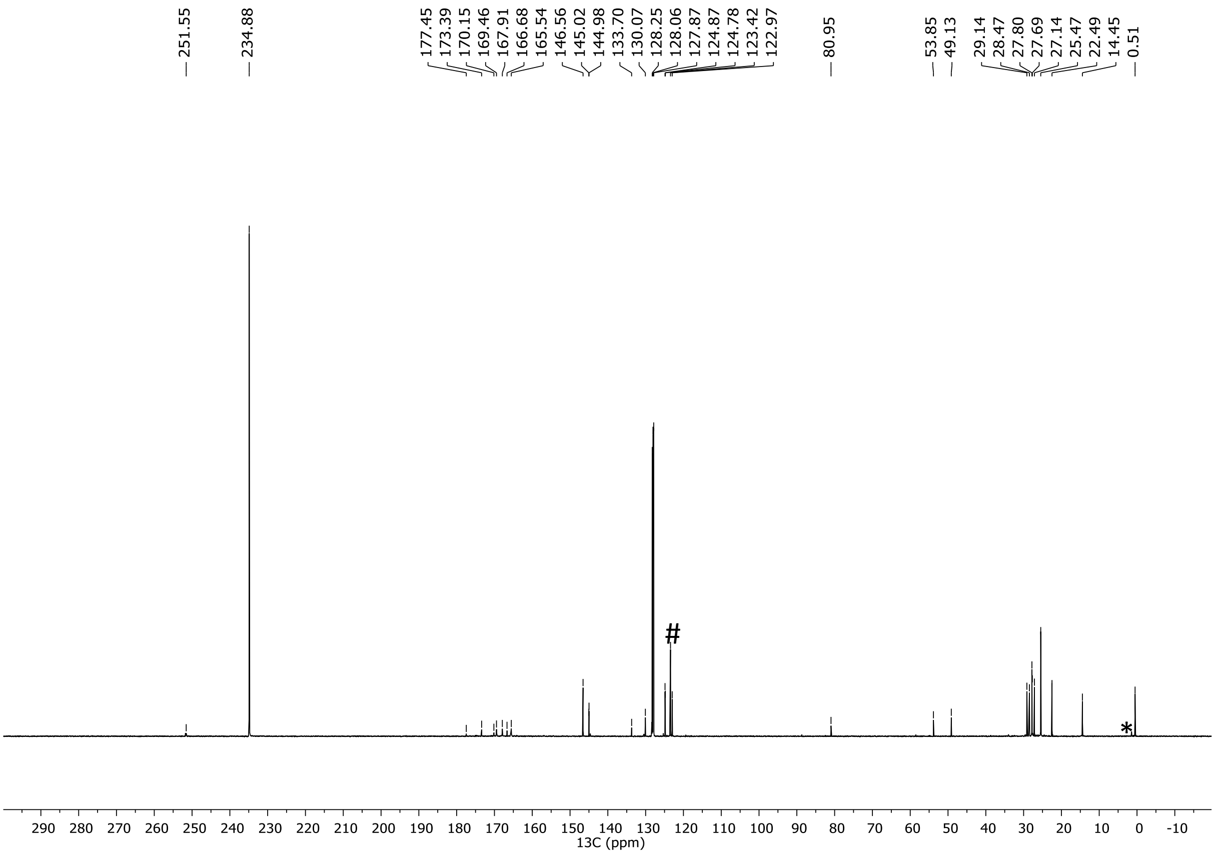

Figure S28. ^{1}H-^{1}H COSY NMR Spectrum of **6**.

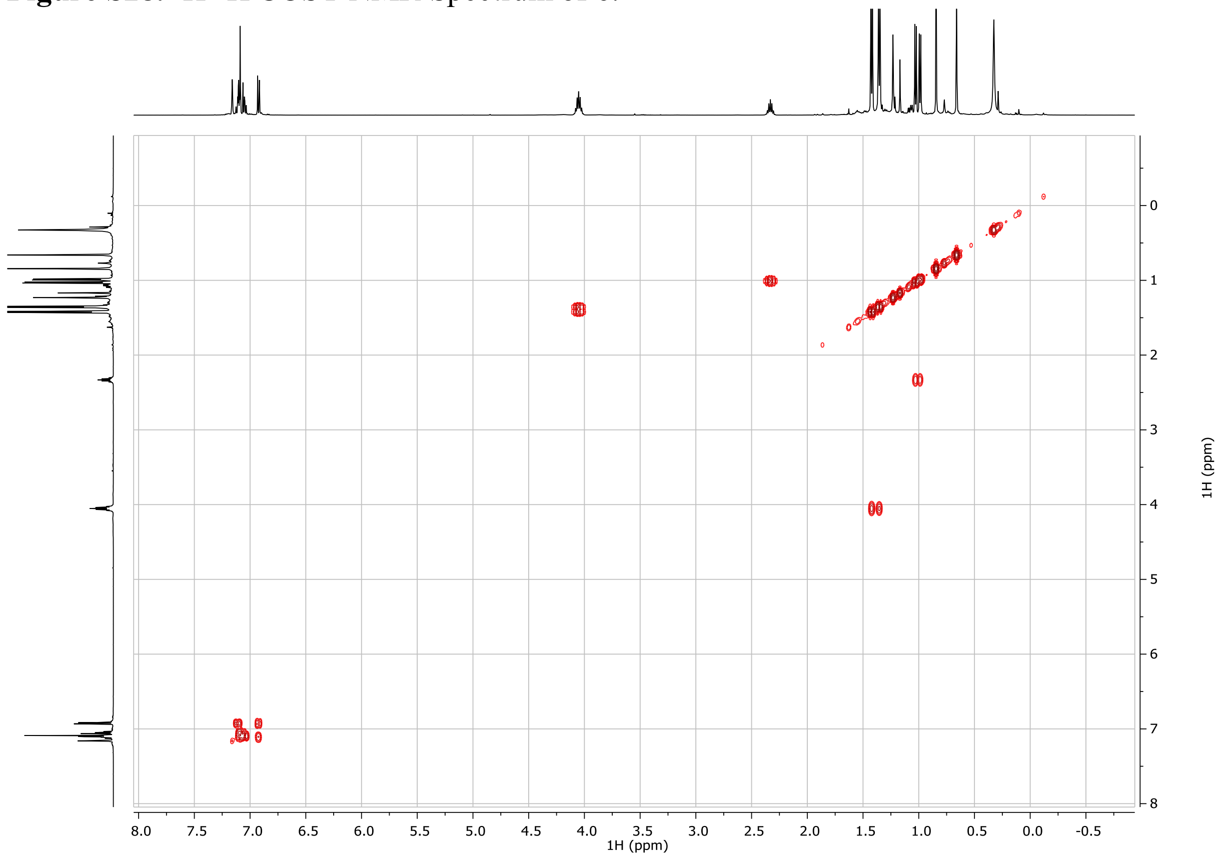

Figure S29. ^{1}H-^{13}C HSQC NMR Spectrum of **6**.

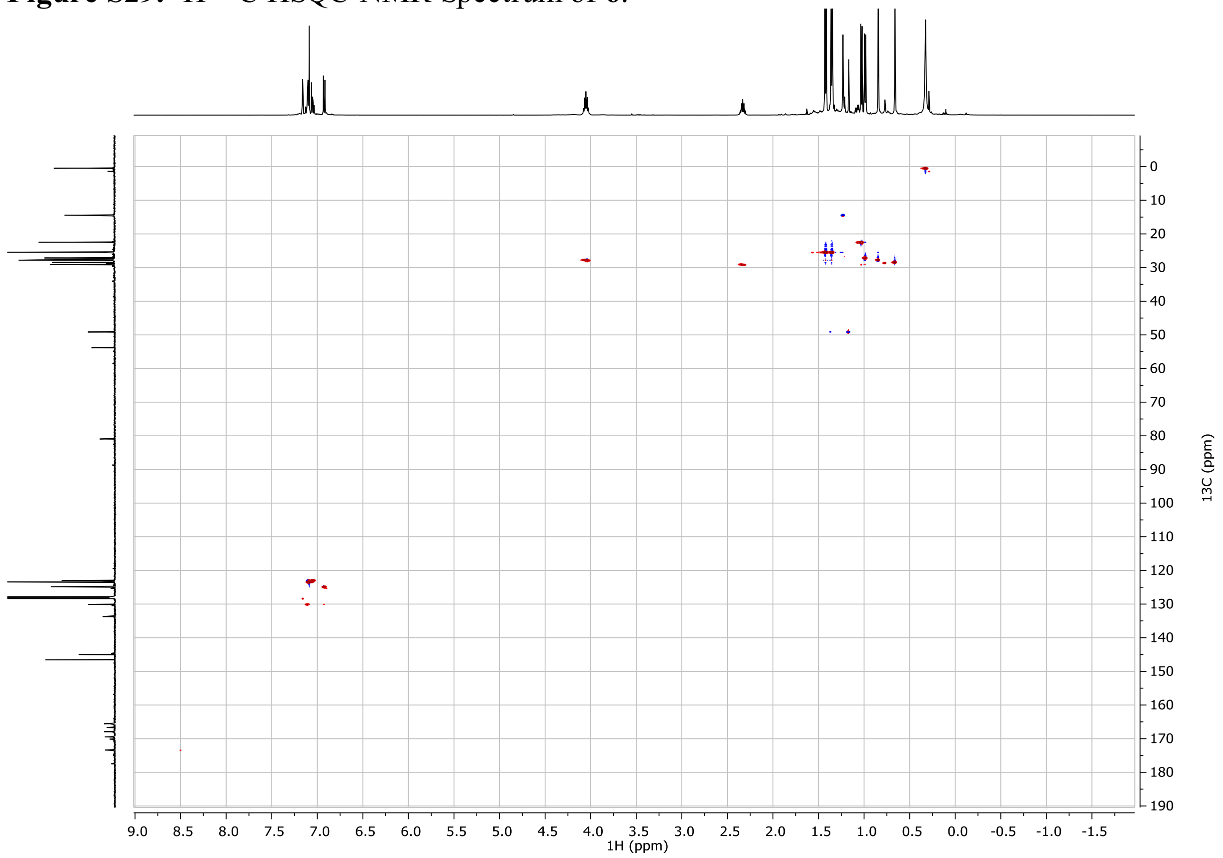

Figure S30. ^1H-^{13}C HMBC NMR Spectrum of **6**.

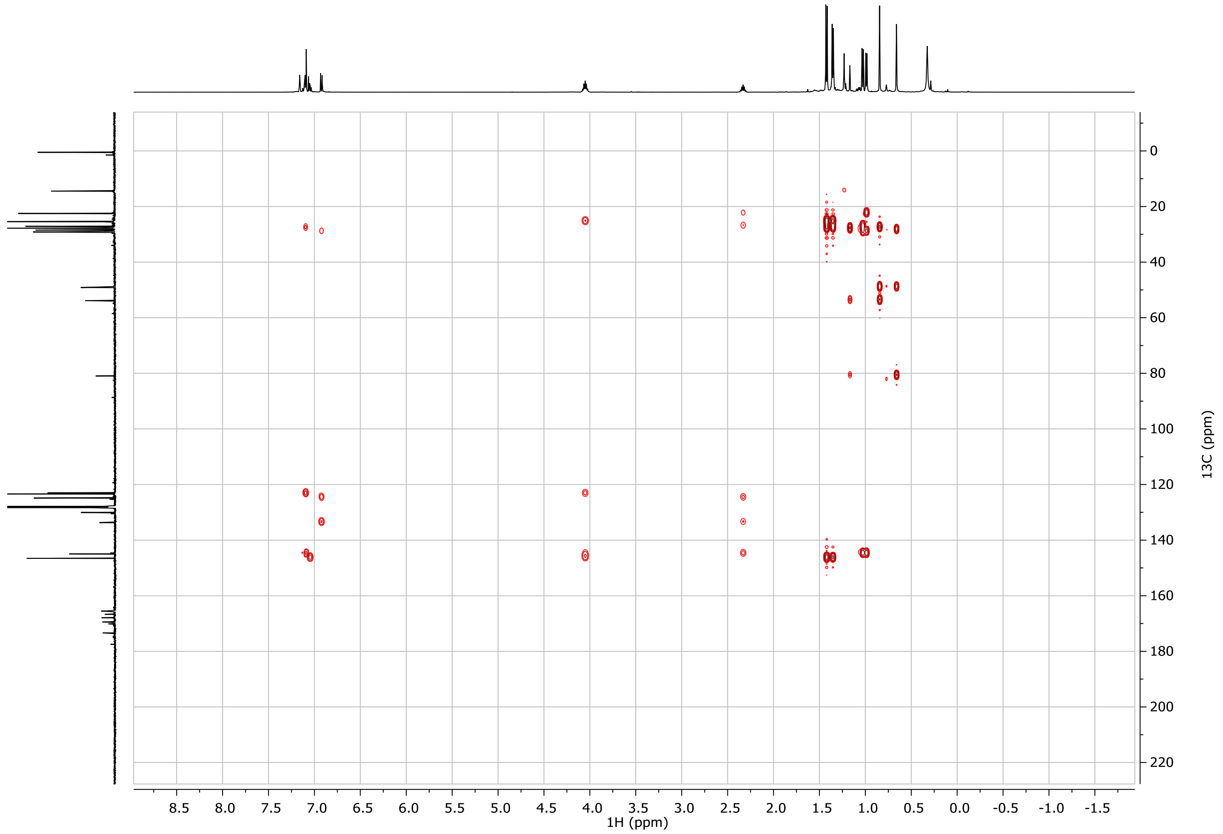

Single Crystal X-ray Diffraction Analysis

Data were collected for compounds **1** - **4** and **6** on a SuperNova, Dual Cu at zero, EosS2 diffractometer (Cu*K*α; λ = 1.54184 Å). The crystals were all kept at 150(2) K during data collection. Using Olex2,[5] the structure was solved with the ShelXT[6] structure solution program using Intrinsic Phasing and refined with the ShelXL[7] refinement package using Least Squares minimization.

There is one guest molecule of methyl cyclohexane in the asymmetric unit of compound **1**.

The asymmetric unit in **3** comprises one molecule of the bimetallic complex and a complete hexane molecule with half site-occupancy. The latter is disordered about a crystallographic inversion centre and has been refined with the inclusion of distance and ADP restraints, to assist convergence. The ADPs indicate additional smearing of electron density along the length of the hexane moiety but this was not modelled.

The asymmetric unit on **4** was seen to contain 1 molecule of the metal complex and one molecule of hexane. While the latter was readily identifiable, it was disordered to an extent that did not lend itself to credible modelling and, as such, guest solvent was addressed using the solvent mask algorithm in Olex-2. The presence of solvent has been accounted for in the formula as presented. C33, C34, C35, C37 and C38 were each modelled over 2 sites, in a 50:50 ratio. ADP and distance restraints were included, for fractional occupancy carbons, to assist convergence.

Table S1: Single crystal X-ray diffraction analysis of compounds **1 – 4** and **6**.

Compound	**1**	**2**	**3**	**4**	**6**
Empirical formula	$C_{48}H_{84}AlCuN_4Si_2$	$C_{50}H_{81}AlCuN_3Si_2$	$C_{51}H_{91}AlCuN_6Si_2$	$C_{63}H_{109}AlCuN_5Si_2$	$C_{51}H_{81}AlCuN_3O_2Si_2$
Formula weight	863.89	870.87	934.99	1083.25	914.88
Crystal system	monoclinic	orthorhombic	triclinic	monoclinic	monoclinic
Space group	$P2_1/n$	$P2_12_12_1$	P-1	$P2_1$	$P2_1$
a/Å	9.8859(1)	13.5166(1)	9.9857(3)	16.7744(1)	12.4775(1)
b/Å	18.7781(1)	19.1988(1)	16.5218(5)	10.1320(1)	16.0721(1)
c/Å	27.8353(2)	19.7555(1)	17.1986(4)	19.3730(2)	14.3837(1)
α/°	90	90	90.874(2)	90	90
β/°	96.8140(10)	90	90.617(2)	101.104(1)	111.948(1)
γ/°	90	90	104.471(3)	90	90
U/Å^3	5130.80(7)	5126.60(5)	2746.80(14)	3230.96(5)	2675.44(4)
Z	4	4	2	2	2
ρ_{calc}g cm^{-3}	1.118	1.128	1.130	1.113	1.136
μ/mm^{-1}	1.457	1.459	1.406	1.252	1.450
F(000)	1880.0	1888.0	1018.0	1184.0	988.0
Crystal size/mm^3	0.293 × 0.202 × 0.149	0.241 × 0.209 × 0.183	0.286 × 0.24 × 0.136	0.24 × 0.14 × 0.078	0.211 × 0.174 × 0.098
2θ range for data collection/°	5.69 to 146.308	6.42 to 143.34	5.14 to 146.412	5.368 to 144.258	6.626 to 142.634
Index ranges	-11 ≤ h ≤ 12, -20 ≤ k ≤ 23, -34 ≤ l ≤ 34	-16 ≤ h ≤ 16, -23 ≤ k ≤ 20, -24 ≤ l ≤ 24	-11 ≤ h ≤ 12, -19 ≤ k ≤ 20, -20 ≤ l ≤ 21	-20 ≤ h ≤ 20, -12 ≤ k ≤ 12, -22 ≤ l ≤ 23	-15 ≤ h ≤ 10, -19 ≤ k ≤ 19, -17 ≤ l ≤ 17
Reflections collected	67476	61131	35272	68468	31439
Independent reflections, R_{int}	10232 [R_{int} = 0.0266, R_{sigma} = 0.0171]	10028 [R_{int} = 0.0434, R_{sigma} = 0.0263]	10974 [R_{int} = 0.0313, R_{sigma} = 0.0295]	12722 [R_{int} = 0.0463, R_{sigma} = 0.0308]	9949 [R_{int} = 0.0383, R_{sigma} = 0.0386]
Data/restraints/parameters	10232/0/525	10028/0/534	10974/41/601	12722/36/667	9949/1/561
Goodness−of−fit on F^2	1.031	1.026	1.040	1.064	1.035
Final R1, wR_2[I>=2σ (I)]	R_1 = 0.0294, wR_2 = 0.0759	R_1 = 0.0274, wR_2 = 0.0748	R_1 = 0.0435, wR_2 = 0.1208	R_1 = 0.0397, wR_2 = 0.1052	R_1 = 0.0283, wR_2 = 0.0734
Final R1, wR_2[all data]	R_1 = 0.0314, wR_2 = 0.0777	R_1 = 0.0282, wR_2 = 0.0755	R_1 = 0.0467, wR_2 = 0.1242	R_1 = 0.0412, wR_2 = 0.1068	R_1 = 0.0290, wR_2 = 0.0741
Largest diff. peak/hole / e Å^{-3}	0.26/-0.24	0.26/-0.17	0.92/-0.50	0.51/-0.27	0.37/-0.35
Flack parameter	-	-0.015(7)	-	-0.021(16)	0.011(8)

Computational Details / Methodology

DFT calculations were run with Gaussian 09 (Revision D.01).[8] The Al and Cu centres were described with the Stuttgart RECPs and associated basis sets,[9] and 6-31G** basis sets were used for all other atoms (BS1).[10] A polarization function was also added to Al (ζd = 0.180). Initial BP86[11] optimizations were performed using the 'grid = ultrafine' option, with all stationary points being fully characterized via analytical frequency calculations as minima (all positive eigenvalues). The BP86-optimized geometries of **1** and **2** were used for NBO (Natural Bond Orbital) studies.

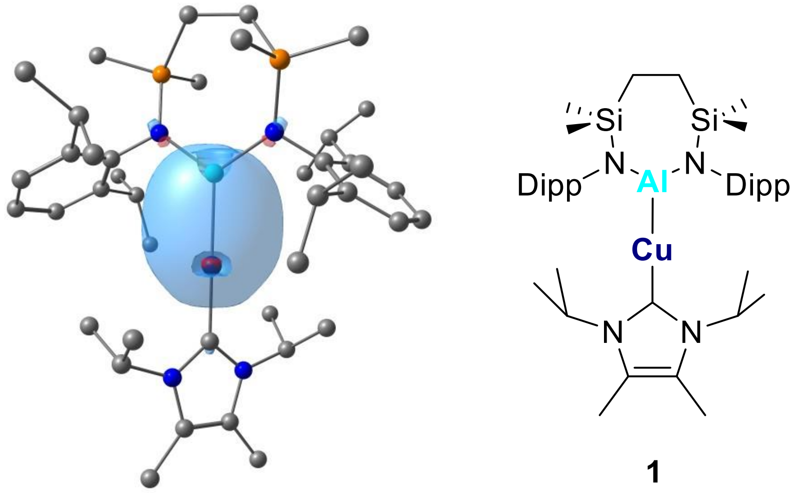

1

$\sigma_{Al—Cu}$ = 0.600Al + 0.400Cu

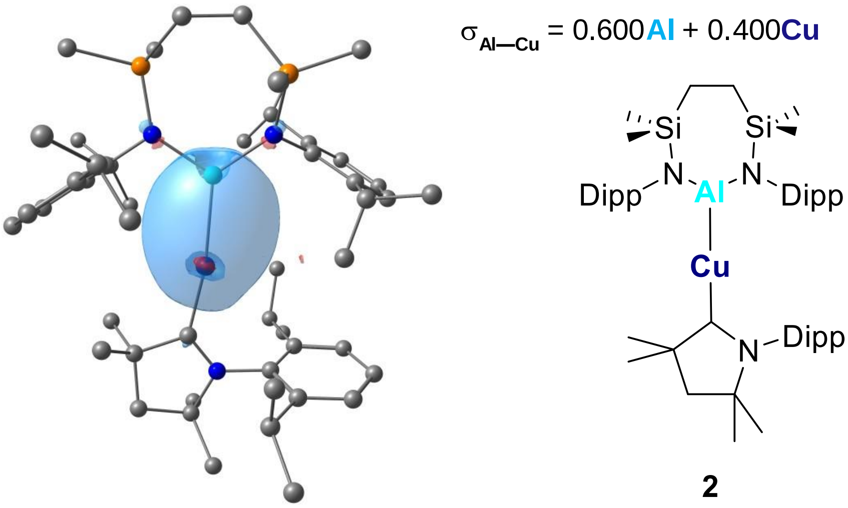

2

Figure S31. Localized molecular orbitals of **1** and **2** showing the Al–Cu σ-bonding orbital.

Table S2: Cartesian Coordinates and Computed Energies (in Hartrees)

1

SCF (BP86) Energy = -2031.04455887

Enthalpy 0K = -2030.039962

Enthalpy 298K = -2029.974554

Free Energy 298K = -2030.140763

Lowest Frequency = 15.1372 cm^{-1}

Second Frequency = 22.0353 cm^{-1}

SCF (BP86-D3BJ) Energy = -2031.36231087

SCF (Toluene) Energy = -2031.04885038

SCF (BS2) Energy = -2843.16767247

```
Cu -1.54060 -0.00144  0.00121
Si  3.53488  1.66066 -1.05142
Si  3.53761 -1.65643  1.05041
Al  0.84199  0.00030  0.00017
N   1.88148  1.53819 -0.35909
N   1.88375 -1.53621  0.35879
N  -4.35626 -0.83627 -0.68706
N  -4.35723  0.83118  0.68862
C   1.16847  2.78267 -0.19773
C   0.40246  3.34893 -1.26741
C  -0.26330  4.57533 -1.06737
H  -0.84049  5.00684 -1.89417
C  -0.20268  5.25433  0.15442
H  -0.72102  6.21069  0.28541
C   0.53456  4.69577  1.20575
H   0.58568  5.22233  2.16592
C   1.22457  3.47824  1.05366
C   0.27724  2.67012 -2.63250
H   0.85911  1.73406 -2.58129
C  -1.18628  2.28894 -2.94651
H  -1.59275  1.62448 -2.16142
H  -1.25246  1.76117 -3.91514
H  -1.83433  3.18210 -3.00602
C   0.86410  3.53768 -3.76915
H   0.31029  4.48740 -3.87816
H   0.80380  3.00582 -4.73568
H   1.92173  3.79091 -3.58481
C   2.00335  2.90913  2.23972
H   2.65852  2.11899  1.83321
C   1.04439  2.23933  3.25042
H   0.33574  2.97783  3.66707
H   1.60136  1.78812  4.09130
H   0.44967  1.44432  2.76493
C   2.89762  3.95054  2.94457
H   3.57878  4.44768  2.23324
H   3.51176  3.46380  3.72261
H   2.30474  4.73752  3.44408
C   4.05771  3.49140 -1.14489
H   3.33086  4.10583 -1.69999
H   5.03468  3.57227 -1.65315
H   4.16167  3.93254 -0.13933
C   3.62222  0.91431 -2.80835
H   3.22322 -0.11366 -2.83363
H   4.66988  0.87525 -3.15716
H   3.04879  1.51508 -3.53331
C   4.86593  0.77544 -0.00178
H   5.82675  1.15001 -0.41350
H   4.82306  1.16829  1.03208
C   4.86704 -0.76932  0.00030
H   5.82853 -1.14249  0.41173
H   4.82443 -1.16225 -1.03354
C   3.62451 -0.91003  2.80735
H   3.22379  0.11727  2.83288
H   4.67220 -0.86926  3.15585
H   3.05231 -1.51184  3.53243
C   4.06295 -3.48646  1.14374
H   3.33780 -4.10153  1.70033
H   5.04085 -3.56582  1.65047
H   4.16583 -3.92797  0.13824
C   1.17242 -2.78166  0.19754
C   0.40763 -3.34913  1.26745
C  -0.25655 -4.57639  1.06748
```

H -0.83282 -5.00881 1.89445
C -0.19551 -5.25511 -0.15444
H -0.71261 -6.21216 -0.28535
C 0.54054 -4.69540 -1.20598
H 0.59200 -5.22175 -2.16625
C 1.22894 -3.47695 -1.05399
C 0.28203 -2.67067 2.63267
H 0.86276 -1.73391 2.58141
C -1.18184 -2.29126 2.94716
H -1.58932 -1.62716 2.16228
H -1.24837 -1.76370 3.91587
H -1.82883 -3.18519 3.00672
C 0.87026 -3.53769 3.76902
H 0.31768 -4.48814 3.87799
H 0.80953 -3.00609 4.73567
H 1.92816 -3.78956 3.58438
C 2.00644 -2.90656 -2.24027
H 2.66073 -2.11564 -1.83387
C 1.04611 -2.23783 -3.25039
H 0.33827 -2.97720 -3.66687
H 1.60208 -1.78568 -4.09143
H 0.45052 -1.44372 -2.76447
C 2.90177 -3.94661 -2.94575
H 3.58398 -4.44294 -2.23485
H 3.51486 -3.45889 -3.72400
H 2.30974 -4.73432 -3.44511
C -3.50886 -0.00247 0.00130
C -3.89497 -1.93346 -1.57954
H -4.81945 -2.38594 -1.97680
C -3.08124 -1.37381 -2.75570
H -3.65010 -0.60932 -3.31030
H -2.81763 -2.18970 -3.44899
H -2.14466 -0.91613 -2.38944
C -3.12688 -2.99822 -0.78425
H -2.19196 -2.58192 -0.36969
H -2.85546 -3.83971 -1.44194
H -3.73373 -3.38759 0.04988
C -5.70228 -0.53453 -0.43887
C -6.85211 -1.27477 -1.04861
H -6.85459 -1.21140 -2.15176
H -7.80331 -0.84720 -0.69560
H -6.85369 -2.34561 -0.77622
C -5.70291 0.52964 0.43829
C -6.85357 1.26978 1.04656
H -6.85790 1.20573 2.14967
H -7.80429 0.84264 0.69171
H -6.85445 2.34077 0.77483
C -3.89717 1.92879 1.58123
H -4.82213 2.37902 1.97995
C -3.13231 2.99558 0.78551
H -2.19706 2.58140 0.36964
H -2.86185 3.83735 1.44325
H -3.74101 3.38407 -0.04769
C -3.08081 1.37029 2.75607
H -3.64732 0.60426 3.31094
H -2.81819 2.18643 3.44944
H -2.14362 0.91487 2.38852

2

SCF (BP86) Energy = -2325.83210875

Enthalpy 0K = -2324.660822

Enthalpy 298K = -2324.587675

Free Energy 298K = -2324.767693

Lowest Frequency = 11.7557 cm^{-1}

Second Frequency = 18.7026 cm^{-1}

SCF (BP86-D3BJ) Energy = -2326.21387351

SCF (Toluene) Energy = -2325.83611982

SCF (BS2) Energy = -3138.01898184

Cu 0.90298 -0.64424 0.07948
Si -4.59322 -1.00760 -0.03713
Si -3.39392 2.39806 1.50353
Al -1.39964 0.12777 0.18311
N -2.80065 -1.15422 0.11429
N -2.04460 1.89521 0.43378
N 3.85253 -1.01572 -0.28806

C	-2.31657	-2.51575	0.10133
C	-2.07484	-3.20098	-1.13351
C	-1.61149	-4.53194	-1.10728
H	-1.43457	-5.05178	-2.05664
C	-1.37961	-5.20510	0.09758
H	-1.02554	-6.24184	0.09612
C	-1.61533	-4.53707	1.30535
H	-1.44261	-5.06211	2.25206
C	-2.08512	-3.20939	1.33398
C	-2.32085	-2.53820	-2.49106
H	-2.76089	-1.54838	-2.28784
C	-3.31951	-3.34059	-3.35519
H	-2.91076	-4.32711	-3.63842
H	-3.54665	-2.79814	-4.29029
H	-4.26918	-3.51791	-2.82261
C	-1.00905	-2.29911	-3.27066
H	-0.31456	-1.66803	-2.68832
H	-1.21513	-1.79014	-4.22932
H	-0.49631	-3.25100	-3.49958
C	-2.33164	-2.53843	2.68664
H	-2.91412	-1.62295	2.48075
C	-3.15066	-3.41323	3.66004
H	-2.59323	-4.31340	3.97512
H	-4.09820	-3.75063	3.20718
H	-3.39219	-2.84381	4.57477
C	-1.00034	-2.10582	3.34326
H	-1.18115	-1.57157	4.29356
H	-0.42504	-1.44006	2.67285
H	-0.36763	-2.98521	3.56213
C	-5.40428	-2.69495	0.32848
H	-5.01425	-3.49621	-0.31960
H	-6.49325	-2.61932	0.16077
H	-5.24474	-3.00616	1.37418
C	-5.19063	-0.47277	-1.77465
H	-4.67553	0.42880	-2.14308
H	-6.27047	-0.24216	-1.72584
H	-5.05520	-1.26996	-2.52286
C	-5.36512	0.23372	1.19109

H	-6.45335	0.02573	1.11651
H	-5.08672	-0.05514	2.22230
C	-5.10065	1.73626	0.95081
H	-5.83328	2.34312	1.52299
H	-5.26747	2.00212	-0.11035
C	-3.13037	1.75166	3.28531
H	-2.88265	0.67596	3.28855
H	-4.05100	1.88114	3.88227
H	-2.31568	2.28260	3.80488
C	-3.55010	4.29620	1.52036
H	-2.63819	4.79613	1.88116
H	-4.38431	4.58825	2.18226
H	-3.76502	4.68636	0.51140
C	-1.31018	2.93297	-0.24945
C	-1.56859	3.20771	-1.63086
C	-0.82733	4.20406	-2.29692
H	-1.03747	4.40511	-3.35442
C	0.15624	4.94684	-1.63669
H	0.71896	5.72276	-2.16764
C	0.39935	4.69471	-0.28064
H	1.16199	5.28042	0.24474
C	-0.31592	3.71200	0.42901
C	-2.65854	2.47153	-2.40728
H	-3.12311	1.76535	-1.69799
C	-2.08323	1.65269	-3.58328
H	-1.59290	2.30588	-4.32747
H	-2.88413	1.09660	-4.10274
H	-1.33147	0.92421	-3.23343
C	-3.76232	3.43493	-2.89913
H	-4.20359	4.00164	-2.06171
H	-4.57372	2.87622	-3.39934
H	-3.36651	4.16782	-3.62482
C	0.00722	3.48297	1.90445
H	-0.84392	2.93448	2.33950
C	1.24794	2.58098	2.06221
H	1.09952	1.61431	1.54462
H	1.44810	2.37563	3.12917
H	2.14403	3.05830	1.62868

C	0.19151	4.78758	2.70932
H	1.10413	5.33277	2.40912
H	0.29198	4.55915	3.78509
H	-0.66184	5.47593	2.58537
C	2.64336	-1.54045	-0.11230
C	2.76504	-3.06524	-0.23178
C	2.15664	-3.71554	1.03323
H	2.30264	-4.81008	0.99364
H	1.07473	-3.51723	1.10197
H	2.64081	-3.33836	1.95078
C	1.99252	-3.56928	-1.47372
H	2.35995	-3.11646	-2.40987
H	0.91914	-3.34421	-1.38041
H	2.10432	-4.66487	-1.55659
C	4.29859	-3.33885	-0.32447
H	4.68304	-3.65053	0.66341
H	4.53777	-4.14414	-1.03865
C	4.95808	-2.00569	-0.73408
C	6.30767	-1.75556	-0.04563
H	6.68142	-0.73689	-0.23973
H	7.04535	-2.46715	-0.45364
H	6.26046	-1.91453	1.04131
C	5.16239	-1.89288	-2.25910
H	4.23337	-2.08150	-2.81874
H	5.90728	-2.64311	-2.57486
H	5.54787	-0.89989	-2.54021
C	4.15368	0.39616	-0.08070
C	4.61667	0.78863	1.20815
C	4.97493	2.13745	1.39167
H	5.33315	2.46536	2.37261
C	4.86727	3.06794	0.35111
H	5.15590	4.11137	0.51585
C	4.36472	2.66682	-0.89014
H	4.24197	3.40685	-1.68717
C	3.99349	1.32943	-1.13859
C	4.67228	-0.16723	2.40531
H	4.58368	-1.19826	2.02433
C	3.46633	0.05677	3.34638
H	3.47297	1.07979	3.75994
H	3.50326	-0.65347	4.19133
H	2.51219	-0.08591	2.81304
C	5.99672	-0.06891	3.19385
H	6.87794	-0.19553	2.54294
H	6.03062	-0.84720	3.97566
H	6.09585	0.90461	3.70411
C	3.36829	0.97827	-2.48921
H	3.26216	-0.11702	-2.54341
C	1.94617	1.57429	-2.59355
H	1.29319	1.19067	-1.78760
H	1.48890	1.30449	-3.56159
H	1.95527	2.67442	-2.51446
C	4.23788	1.43084	-3.68337
H	4.30699	2.53089	-3.74083
H	3.79092	1.08283	-4.63037
H	5.26736	1.03700	-3.62573

References

1. R. J. Schwamm, M. P. Coles, M. S. Hill, M. F. Mahon, C. L. McMullin, N. A. Rajabi, A. S. S. Wilson, *Angew. Chem. Int. Ed.* **2020**, *59*, 3928.
2. N. Kuhn, Kratz, *Synthesis* **1993**, *6*, 561.
3. V. Lavallo, Y. Canac, C. Präsang, B. Donnadieu, G. Bertrand, *Angew. Chem. Int. Ed.* **2005**, *44*, 5705.
4. A. s. Romanov, C. R. Becker, C. E. James, D. Di, D. Credgington, M. Linnolahti, M. Bochmann, *Chem. Eur. J.* **2017**, *23*, 4625.
5. O. V. Dolomanov, L. J. Bourhis, T. J. Gildea, J. A. K. Howard, H. Puschmann, *J. Appl. Cryst.* **2009**, *42*, 339-341.
6. G. M. Sheldrick, *Acta Cryst.* **2015**, *A71*, 3-8.
7. G. M. Sheldrick, *Acta Cryst.* **201**, *C71*, 3-8.
8. Frisch, M. J.; Trucks, G. W.; Schlegel, H. B.; Scuseria, G. E.; Robb, M. A.; Cheeseman, J. R.; Scalmani, G.; Barone, V.; Mennucci, B.; Petersson, G. A.; Nakatsuji, H.; Caricato, M.; Li, X.; Hratchian, H. P.; Izmaylov, A. F.; Bloino, J.; Zheng, G.; Sonnenberg, J. L.; Hada, M.; Ehara, M.; Toyota, K.; Fukuda, R.; Hasegawa, J.; Ishida, M.; Nakajima, T.; Honda, Y.; Kitao, O.; Nakai, H.; Vreven, T.; Montgomery, J. A., Jr.; Peralta, J. E.; Ogliaro, F.; Bearpark, M.; Heyd, J. J.; Brothers, E.; Kudin, K. N.; Staroverov, V. N.; Kobayashi, R.; Normand, J.; Raghavachari, K.; Rendell, A.; Burant, J. C.; Iyengar, S. S.; Tomasi, J.; Cossi, M.; Rega, N.; Millam, J. M.; Klene, M.; Knox, J. E.; Cross, J. B.; Bakken, V.; Adamo, C.; Jaramillo, J.; Gomperts, R.; Stratmann, R. E.; Yazyev, O.; Austin, A. J.; Cammi, R.; Pomelli, C.; Ochterski, J. W.; Martin, R. L.; Morokuma, K.; Zakrzewski, V. G.; Voth, G. A.; Salvador, P.; Dannenberg, J. J.; Dapprich, S.; Daniels, A. D.; Farkas, O.; Foresman, J. B.; Ortiz, J. V.; Cioslowski, J.; Fox, D. J. Gaussian 09 (Revision D.01); Gaussian Inc.: Wallingford, CT, 2009.
9. D. Andrae, U. Häußermann, M. Dolg, H. Stoll, H. Preuß, *Theor. Chim. Acta*, **1990**, *77*, 123−141

(1990).

10. (a) P. C. Hariharan, J. A. Pople, *Theor. Chim. Acta*, **1973**, *28*, 213−222. (b) W. J. Hehre, R. Ditchfield, J. A. Pople, J. A. *J. Chem. Phys*. **1972**, *56*, 2257.

11. (a) A. D. Becke, *Phys. Rev. A: At., Mol., Opt. Phys.* **1988**, *38*, 3098. (b) J. P. Perdew, *Phys. Rev. B: Condens. Matter Mater. Phys.* **1986**, *33*, 8822−8824.
